# Supplementary material for: Psychometric validation of the education and assessment of genetic literacy or the EAGL measure
Source: HGG Adv. 2026 Jul 8;7(4):100651. doi: 10.1016/j.xhgg.2026.100651 (PMC13396853; doi:10.1016/j.xhgg.2026.100651)
Supplement: Document S1. Figure S1 and Tables S1–S11 [file mmc1.pdf]

HGGA, Volume 7

Supplemental information

Psychometric validation of the education and assessment of genetic literacy or the EAGL measure

Lily S. Barna, Yi Liao, Michael R. Wierzbicki, Gabriela M. Ramírez-Renta, Kimberly A. Kaphingst, and Chris Gunter

Supplementary Information Guide
Barna LS et al, “Psychometric Validation of the Education and Assessment of Genetic Literacy (EAGL) Measure”

Figure S1: Exploratory Factor Analysis (EFA) results for the EAGL-long, 3, 4, 5, and 9-factor solutions

Table S1. Participant Characteristics for Combined Sample (N = 2708), Split by Sample

Table S2: Comparison of Mean EAGL-Short Scores Across Three Survey Runs

Table S3: Complete Evaluation and Assessment of Genetic Literacy (EAGL-long) Instrument

Table S4: Factor Loadings and Communalities (h^2) for EAGL-long (Exploratory Factor Analysis, EFA)

Table S5: Five Factor EFA Table

Table S6. Four Factor EFA Table

Table S7: Eigenvalues and Variance Explained for Exploratory Factor Analysis of the EAGL-long

Table S8: Descriptive Statistics for Subjective Knowledge Items

Table S9: Frequency distribution for Knowledge Comprehension and Conceptual Knowledge items in CFA Sample (EAGL3, n = 1001)

Table S10: Average Scores Per Subscale for EAGL-long and EAGL-short

Table S11. Adjusted Mean Estimates for Each Subscale in EAGL-short

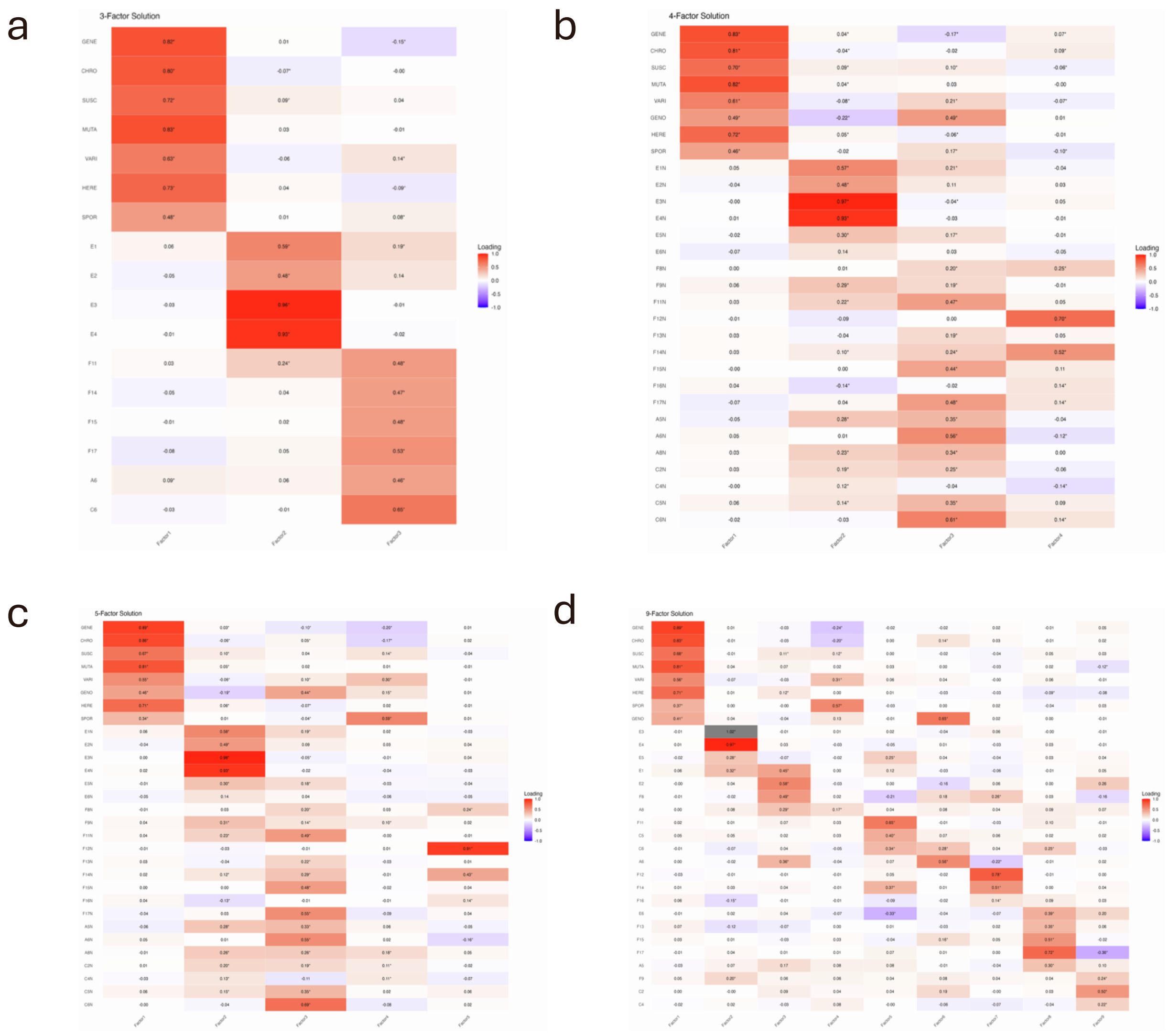

Figure S1. Exploratory Factor Analysis (EFA) results for the EAGL-long, 3, 4, 5, and 9-factor solutions. The heatmap displays factor loadings, with color intensity representing the magnitude of loadings (red for positive, blue for negative), and asterisks (*) indicating statistically significant loadings. Questions are grouped to highlight primary factor associations, with some items loading on multiple factors significantly, or loading on none. **(a)** 3-factor solution. **(b)** 4-factor solution. **(c)** 5-factor solution. **(d)** 9-factor solution contains nuanced splicing of clusters which illustrate multiple subject domains being tested. Loading values represent the strength of association between each item and the factor.

Table S1. Participant Characteristics for Combined Sample (N = 2708), Split by Sample				
Sample	Variable	Category	Frequency	% Total
Sample 1 (N = 1005)	Age	26-39	249	24.8%
		50-59	217	21.6%
		40-49	179	17.8%
		60-69	175	17.4%
		18-25	136	13.5%
		70+	49	4.9%
	Education	Bachelor's degree	398	39.6%
		High school diploma or equivalent	275	27.4%
		Professional degree	159	15.8%
		Associate degree	138	13.7%
		Doctorate degree	24	2.4%
		Some high school, no diploma	9	0.9%
		Kindergarten through 8th grade	2	0.2%
	Connection to Autism	No	822	81.8%
		Yes	183	18.2%
	Metro Status	Metro	896	89.15%
		Nonmetro	108	10.75
		Invalid Responses	1	0.10%
Sample 2 (n = 702)	Variable	Category	Frequency	% Total
	Age	18-25	134	19.1
		26-39	282	40.2
		40-49	142	20.2
		50-59	91	13.0
		60-69	43	6.1
		70+	10	1.4
	Education	High school diploma or equivalent	379	54.0%
		Associate degree	145	20.7%
		Bachelor's degree	95	13.5%
		Professional degree	41	5.8%
		Doctorate degree	22	3.1%
		Some high school, no diploma	18	2.6%
		Kindergarten through 8th grade	2	0.3%
	Connection to Autism	No	523	74.5%
		Yes	179	25.5%
	Metro Status	Metro	598	85.19%
		Nonmetro	102	14.53%
		Invalid Responses	2	0.28%

Sample 3 (n = 1001)	Variable	Category	Frequency	% Total
	Age	26-39	431	43.0%
		40-49	217	21.7%
		18-25	152	15.2%
		50-59	128	12.8%
		60-69	54	5.4%
		70+	19	1.9%
	Education	High school diploma or equivalent	441	44.0%
		Bachelor's degree	292	29.2%
		Associate degree	124	12.4%
		Professional degree	111	11.1%
		Doctorate degree	18	1.8%
		Some high school, no diploma	14	1.4%
		Kindergarten through 8th grade	1	0.1%
	Connection to Autism	No	791	79.0%
		Yes	210	21.0%
	Metro Status	Metro	880	87.91%
		Nonmetro	120	11.99%
		Invalid Responses	1	0.10%

Table S1. The table presents demographic characteristics across three EAGL samples comprising a total of 2,708 participants (Sample 1: N = 1,005; Sample 2: N = 702; Sample 3: N = 1,001). Age categories are presented in years and grouped into six ranges from 18-25 to 70+. Education levels are categorized from elementary education through doctoral degrees. "Connection to Autism" indicates whether participants reported having a personal connection to autism, either through themselves or through family members. Metro/non-metro distinctions were made via Federal Information Processing System (FIPS) Codes for States and Counties and Rural-Urban Continuum Codes (RUCC). A RUCC of 1-3 delineates a metropolitan area, with 4-9 delineating a nonmetropolitan county. The specific numbers indicate the level of adjacency to a metropolitan area, considering population of county and proximity to metro counties. Four participants had to be removed from analysis as they provided invalid 5-digit zip codes. Frequencies represent the number of participants in each category, and percentages are calculated based on the total sample size for each respective sample.

Table S2: Comparison of Mean EAGL-Short Scores Across Three Survey Runs					
Knowledge Domain	EAGL1 Mean (SD)	EAGL2 Mean (SD)	EAGL3 Mean (SD)	Overall Mean (SD)	Coefficient of Variance (CV) (%)
Subjective Knowledge	5.57 (0.98)	5.59 (0.97)	5.56 (0.98)	5.57 (0.97)	0.29
Applied Knowledge	7.05 (1.08)	6.89 (1.13)	6.93 (1.16)	6.96 (1.12)	1.19
Situational Knowledge	5.56 (1.12)	5.53 (1.20)	5.60 (1.19)	5.56 (1.15)	0.57
Knowledge Comprehension	5.47 (0.79)	5.44 (0.82)	5.33 (1.08)	5.41 (0.92)	1.35
Objective Knowledge	14.57 (1.81)	14.30 (1.91)	14.55 (1.88)	14.50 (1.86)	1.04

Table S2. The table presents the comparison of mean EAGL-short scores across the three survey runs. The mean score and standard deviation for each subscale of the EAGL-long are presented, along with the coefficient of variance.

Table S3: Complete Evaluation and Assessment of Genetic Literacy (EAGL-long) Instrument

First, we would like to see how familiar you are with words related to genetics.
For each word below, please rate how familiar you are with the word. For example, marking "Completely familiar" on the scale reflects that you are entirely familiar with the word, while marking "Not at all familiar" on the scale means that you are not familiar with the word in any capacity. Please select the answer that best reflects your view. Following that rating, you will then be presented with a fill-in-the-blank style question that utilizes a word related to genetics. Please do your best to select the appropriate response for the question, and please do not use the Internet or seek outside help in answering the questions.

Not at all familiar			Neither familiar nor unfamiliar			Completely familiar
1	2	3	4	5	6	7

A1_1. Genetic
Genetics is the study of how living things receive common traits from previous ____________.

a. generations **(C)**
b. experiences
c. examinations
d. d. achievements

A1_2. Chromosome
A chromosome contains ______________ material.

a. genetic **(C)**
b. digestive
c. cellular
d. d. brain

A1_3. Susceptibility

Susceptibility to a disease means you _________________ get the disease.

a. eventually will
b. definitely will
c. possibly will (C)
d. d. never will

A1_4 Mutation

A DNA mutation is ______________.

a. a type of cell
b. a type of virus
c. a change in your DNA sequence (C)
d. a measurement of DNA sequence

A1_5. Variation

Having a variation in the genetic code is __________________.

a. always harmful
b. can be harmful or benign (C)
c. always benign
d. extremely rare

A1_6. Genome

Your genome is _______________________.

a. where you make new DNA
b. all of the genes within one chromosome
c. your entire and complete set of DNA (C)
d. where cellular respiration occurs

A1_7. Heredity

Heredity is the transfer of characteristics from ____________________________.

a. the environment to the person
b. the sick to the healthy
c. the biological parent to their child (C)
d. the teacher to their student

A1_8. Sporadic
If someone is diagnosed with breast cancer without _____________ it is considered sporadic.

a. symptoms
b. a tumor
c. those around them knowing
d. a genetically increased likelihood (C)

A2_1. Please select the number 2 (two) from the list of numbers below:

a. 1
b. 4
c. 6
d. 2 (C)

B1_1. Imagine that we rolled a fair, six-sided die 1,000 times. Out of 1,000 rolls, how many times do you think the die would come up even (2, 4, or 6)?

a. 500 **(C)**
b. 250
c. 750

d. 800

B1_2. In the BIG BUCKS LOTTERY, the chances of winning a $10.00 prize is 1%. What is your best guess about how many people would win a $10.00 prize if 1,000 people each buy a single ticket to BIG BUCKS?

a. 100 people out of 1000
b. 50 people out of 1000
c. 10 people out of 1000 **(C)**
d. 1 person out of 1000

B1_3. Which of the following numbers represents the biggest risk of getting a disease?

a. 1 in 100
b. 1 in 10 **(C)**
c. 1 in 1000

C1_1. You are with your biological aunt, cousin, and sibling. You are asked to line them up by the amount of DNA, or genetic material, you share. You order them by most amount of DNA shared to least. That order is:

a. Aunt, cousin, sibling
b. Sibling, aunt, cousin **(C)**
c. Sibling, cousin, aunt
d. Cousin, sibling, aunt

C1_2. If both of my biological parents have brown eyes, what eye color could I have?

a. Only brown eyes
b. Brown or blue eyes
c. Brown, blue, or green eyes **(C)**
d. Only green eyes

C1_3. Certain genetic variants in the *BRCA* genes increase someone's risk for developing breast cancer. What is the most common purpose for obtaining *BRCA* genetic testing?

a. To determine if someone already has developed breast cancer
b. To determine if someone will never develop breast cancer
c. To determine if someone will one day develop breast cancer due to their environment
d. To determine if someone is genetically predisposed to developing breast cancer **(C)**

C1_4. If you have a genetic disease that was cured in your own red blood cells using gene therapy (replacing defective or missing genes with functional ones) as an adult, can you still pass on the disease to your biological children?

a. Yes, they could still get the disease **(C)**
b. No, they could not get the disease

C1_5. Cloning results in two organisms that are

a. genetically similar
b. genetically identical **(C)**
c. look alike but have different DNA
d. adults

C1_6. The term DNA stands for

a. Dynamic nitrogenous assembly
b. Double-nucleotide association
c. Deoxyribonucleic acid **(C)**
d. Downward nebula artifact

C1_7. When scientists say that a trait is "sex-linked," they mean that the genes related to the trait are

a. On the X or Y chromosome **(C)**
b. On all of the chromosomes
c. Only present in men
d. Only present in women

C2_1. Please select the third option from the list:

a. a
b. b
c. c **(C)**
d. d

D1_1. As far as you know, do you or does anyone in your immediate family have a genetic condition?

Yes ……………………………………………….1
No ……………………………………………….2

D1_2. As far as you know, have you or an immediate family member (e.g. parent, sibling, child) received any form of genetic testing?

Yes ……………………………………………….1
No ……………………………………………….2

[INFOGRAPHIC ABOUT GENETIC AND ENVIRONMENTAL CONTRIBUTORS TO AUTISM DIAGNOSIS HERE]

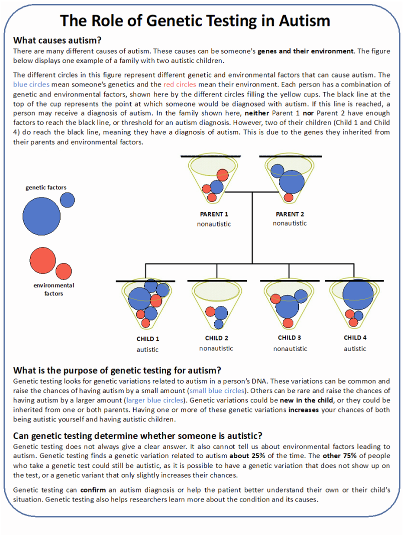

The Role of Genetic Testing in Autism

What causes autism?

There are many different causes of autism. These causes can be someone's **genes and their environment**. The figure below displays one example of a family with two autistic children.

The different circles in this figure represent different genetic and environmental factors that can cause autism. The blue circles mean someone's genetics and the red circles mean their environment. Each person has a combination of genetic and environmental factors, shown here by the different circles filling the yellow cups. The black line at the top of the cup represents the point at which someone would be diagnosed with autism. If this line is reached, a person may receive a diagnosis of autism. In the family shown here, **neither** Parent 1 **nor** Parent 2 have enough factors to reach the black line, or threshold for an autism diagnosis. However, two of their children (Child 1 and Child 4) do reach the black line, meaning they have a diagnosis of autism. This is due to the genes they inherited from their parents and environmental factors.

What is the purpose of genetic testing for autism?

Genetic testing looks for genetic variations related to autism in a person's DNA. These variations can be common and raise the chances of having autism by a small amount (small blue circles). Others can be rare and raise the chances of having autism by a larger amount (larger blue circles). Genetic variations could be **new in the child**, or they could be inherited from one or both parents. Having one or more of these genetic variations **increases** your chances of both being autistic yourself and having autistic children.

Can genetic testing determine whether someone is autistic?

Genetic testing does not always give a clear answer. It also cannot tell us about environmental factors leading to autism. Genetic testing finds a genetic variation related to autism **about 25%** of the time. The **other 75%** of people who take a genetic test could still be autistic, as it is possible to have a genetic variation that does not show up on the test, or a genetic variant that only slightly increases their chances.

Genetic testing can **confirm** an autism diagnosis or help the patient better understand their own or their child's situation. Genetic testing also helps researchers learn more about the condition and its causes.

[CHECKBOXES]
E1. What is the purpose of genetic testing for autism?

Genetic testing always provides a clear answer to whether or not someone is autistic ………..1
Genetic testing can tell you both the genetic and environmental causes of autism …..2
Genetic testing analyzes someone's DNA to find genetic variations related to autism …...3 **(C)**

E2. Please select the phrase that best completes the following statement: Genetic variations that could increase a person's chance of being autistic…

Can be present in some siblings and not others…..1 **(C)**
Are always the exact same between siblings…...2

[NUMERICAL TEXT BOX: RANGE 0-100]
E3. What percentage of individuals who have genetic testing for autism are found to have a variation related to autism?

Enter a number from 0–100: [ ] % **(25)**

[NUMERICAL TEXT BOX: RANGE 0-100]
E4. What percentage of individuals who have genetic testing for autism will receive results with no genetic variations related to autism?

Enter a number from 0–100: [ ] % **(75)**

E5. If neither of the biological parents are autistic, it is impossible for their child to be autistic.

True..1 False...2 **(C)**

E6. It is possible for a child to have a genetic variation that raises their chances of being autistic which neither of their biological parents have.

True..1 **(C)**
False...2

E7. Reaching the threshold for an autism diagnosis is due to a variable amount of environmental and genetic factors that vary per individual.

True..1 **(C)**
False...2

[TRUE/FALSE CHOICES]

**F1_1.** One can see a gene with the naked eye.	F
**F1_2.** Healthy parents can have a child with a hereditary condition.	T
**F1_3.** The onset of certain diseases is due to genes, environment, and lifestyle.	T
**F1_4.** A gene is a disease.	F
**F1_5.** The carrier of a gene linked to a disease may not have the disease themselves.	T
**F1_6.** All serious diseases are hereditary.	F
**F1_7.** Genes control hereditary characteristics.	T
**F1_8.** Genes are inside cells.	T
**F1_9.** The child of a carrier for a genetic condition is always also a carrier for the same condition.	F
**F1_10.** A gene is a piece of DNA.	T
**F1_11.** A gene is a cell.	F
**F1_12.** A gene is a part of a chromosome.	T

**F1_13.** Different genes are expressed in different body parts.	T
**F1_14.** Genes are bigger than chromosomes.	F
**F1_15.** The genome can be changed by human intervention.	T
**F1_16.** It is currently estimated that a person has about 20,000 genes.	T
**F1_17.** Environmental factors, such as UV radiation, can change our DNA sequence.	T

G1_1. What is the most important idea or fact about genetics that you feel you need to know? **[TEXTBOX RESPONSE]**

K1. How old are you?

< 18 1
18 - 25 2
26 - 39 3
40 - 49...4
50 - 59...5
60 - 69 6
70 +...7

K2. What is the highest level of education you have completed?

No schooling completed…..1
Kindergarten through 8th grade..2
Some high school, no diploma 3
High school diploma or equivalent 4
Associate degree...5
Bachelor's degree...6
Professional degree..7
Doctorate degree..8

K3. Are you or is anyone in your immediate family autistic?

Yes 1
No 2

K4. What is the ZIP code of your primary residence? Enter a 5-digit ZIP Code. **[OPEN ENDED BOX]**

K5. Did you answer this survey honestly and to the best of your ability?

Yes 1
No 2

END

Sections:

A1-8 Likert-scale questions measure subjective knowledge
A1-8 multiple choice questions measure applied knowledge
B1-3 questions measure objective numeracy
C1-7 questions measure situational knowledge
E1-7 questions measure knowledge comprehension
F1-17 questions measure objective knowledge
G1 is an optional qualitative comment area
K1-K5 are demographics questions

Questions contained in the EAGL-short measure are shown in Figure 1 of the paper.

Table S4. Factor Loadings and Communalities (h^2) for EAGL-long (Exploratory Factor Analysis)										
Item	Factor1	Factor2	Factor3	Factor4	Factor5	Factor6	Factor7	Factor8	Factor9	h^2
GENE	0.886*	0.011	-0.03	-0.240*	-0.02	-0.022	0.017	-0.007	0.054	0.848
CHRO	0.833*	-0.014	-0.029	-0.204*	0	0.137*	0.034	-0.006	0.023	0.757
SUSC	0.681*	-0.01	0.110*	0.124*	0.002	-0.021	-0.039	0.054	0.029	0.497
MUTA	0.811*	0.037	0.065	0.017	0.028	0	-0.032	0.018	-0.123*	0.681
VARI	0.562*	-0.065	-0.034	0.309*	0.059	0.041	-0.004	0.055	-0.011	0.425
GENO	0.407*	0.035	-0.045	0.132	-0.009	0.648*	0.017	0.002	-0.009	0.607
HERE	0.709*	0.013	0.120*	0.001	0.011	-0.027	-0.026	-0.085	-0.08	0.532
SPOR	0.373*	0.002	-0.004	0.571*	-0.027	0	0.016	-0.042	0.027	0.469
E1	0.06	0.316*	0.448*	0	0.124	-0.027	-0.057	-0.013	0.049	0.326
E2	-0.002	0.038	0.582*	-0.031	0.002	-0.156	0.055	0	0.258	0.435
E3	-0.008	1.017*	-0.008	0.015	0.016	-0.038	0.063	-0.004	-0.01	1.040
E4	0.011	0.973*	0.025	-0.027	-0.049	0.015	-0.026	0.032	-0.005	0.953
E5	-0.024	0.283*	-0.069	-0.022	0.247*	0.039	-0.041	0.025	0.038	0.152
E6	-0.007	0.019	0.045	-0.065	-0.328	-0.039	-0.066	0.393*	0.2	0.315
F8	-0.005	-0.024	0.490*	0.024	-0.212	0.181	0.263*	0.026	-0.161	0.415
F9	0.046	0.195*	0.058	0.064	0.044	0.084	0.041	0.044	0.244*	0.120
F11	0.024	0.01	0.066	0.027	0.645*	-0.01	-0.031	0.096	-0.012	0.432
F12	-0.025	-0.014	-0.008	-0.012	0.052	-0.017	0.776*	-0.015	0.002	0.606
F13	0.066	-0.123	-0.072	0	0.008	-0.033	0.019	0.346*	0.058	0.149
F14	0.014	0.03	0.04	-0.013	0.368*	0.007	0.512*	0	0.037	0.402
F15	0.034	0.01	-0.028	0.027	-0.045	0.162*	0.053	0.512*	-0.024	0.297
F16	0.058	-0.150	-0.008	-0.011	-0.093	-0.022	0.143*	0.093	0.026	0.065
F17	-0.008	0.037	0.006	0.01	0.065	0.014	0.004	0.721*	-0.361*	0.656
A5	-0.03	0.066	0.174	0.08	0.079	-0.014	-0.045	0.300*	0.101	0.151
A6	0.001	-0.02	0.361*	-0.038	0.067	0.561*	-0.219	-0.011	0.023	0.500
A8	0.002	0.079	0.294*	0.171*	0.037	0.078	0.041	0.092	0.072	0.145
C2	0.002	-0.002	0.087	0.035	0.038	0.186	-0.002	0.026	0.503*	0.299
C4	-0.018	0.021	-0.029	0.078	-0.001	-0.064	-0.07	-0.042	0.224*	0.069
C5	0.046	0.05	0.022	0.028	0.399*	0.071	0.06	0.022	0.022	0.175
C6	-0.01	-0.067	0.041	-0.047	0.343*	0.281*	0.041	0.247*	-0.027	0.269

Table S4. This table presents the results of an exploratory factor analysis (EFA) for the EAGL-long instrument, displaying factor loadings and communalities across nine extracted factors (F1-F9). Each row represents a scale question. Factor loadings indicate the correlation between each item and the underlying factors, with values closer to ±1.0 representing stronger relationships. Asterisks (*) denote statistically significant factor loadings. The communality (h^2) represents the proportion of each item's variance explained by the factor solution, with values ranging from 0 to 1, where higher values indicate that more of the item's variance is accounted for by the extracted factors. Survey questions F1-F7 were excluded from this 9-factor solution due to excessively high accuracy rates, suggesting potential issues with model fit or interpretability.

Table S5. Five Factor EFA Table					
Variable	Factor1	Factor2	Factor3	Factor4	Factor5
GENE	0.891*	0.032*	-0.097*	-0.196*	0.007
CHRO	0.857*	-0.058*	0.052*	-0.173*	0.02
SUSC	0.666*	0.100*	0.038	0.142*	-0.035
MUTA	0.812*	0.049*	0.018	0.013	-0.01
VARI	0.546*	-0.059*	0.102*	0.305*	-0.006
GENO	0.455*	-0.194*	0.439*	0.149*	0.007
HERE	0.711*	0.056*	-0.069*	0.019	-0.012
SPOR	0.344*	0.011	-0.037*	0.586*	0.015
E1N	0.058	0.578*	0.187*	0.016	-0.028
E2N	-0.04	0.493*	0.088	0.028	0.044
E3N	0	0.976*	-0.054*	-0.012	0.045
E4N	0.019	0.925*	-0.024	-0.044	-0.028
E5N	-0.011	0.299*	0.181*	-0.032	-0.038
E6N	-0.048	0.138	0.041	-0.058	-0.053
F8N	-0.005	0.032	0.205*	0.025	0.236*
F9N	0.043	0.306*	0.141*	0.098*	0.021
F11N	0.038	0.226*	0.492*	-0.002	-0.009
F12N	-0.012	-0.032	-0.014	0.011	0.906*
F13N	0.033	-0.043	0.216*	-0.026	0.011
F14N	0.021	0.125*	0.290*	-0.011	0.431*
F15N	0.004	0.002	0.479*	-0.018	0.036
F16N	0.045	-0.134*	-0.01	-0.008	0.136*
F17N	-0.045	0.033	0.549*	-0.086	0.035
A5N	-0.063	0.284*	0.331*	0.058	-0.051
A6N	0.054	0.006	0.551*	0.017	-0.157*
A8N	-0.013	0.263*	0.257*	0.181*	0.049
C2N	0.013	0.205*	0.192*	0.108*	-0.022
C4N	-0.026	0.133*	-0.108	0.112*	-0.067
C5N	0.058	0.150*	0.348*	0.021	0.062
C6N	-0.002	-0.039	0.688*	-0.08	0.02

Table S5. Exploratory factor analysis showing factor loadings for EAGL-short scale items across five factors. Asterisks (*) indicate statistically significant loadings. Variables represent different dimensions of genetic knowledge and attitudes, with loadings ranging from -0.196 to 0.976.

Table S6. Four Factor EFA Table				
Variable	Factor1	Factor2	Factor3	Factor4
GENE	0.833*	0.044*	-0.166*	0.073*
CHRO	0.806*	-0.043*	-0.021	0.092*
SUSC	0.699*	0.086*	0.100*	-0.063*
MUTA	0.821*	0.041*	0.025	-0.004
VARI	0.609*	-0.080*	0.214*	-0.065*
GENO	0.488*	-0.216*	0.488*	0.012
HERE	0.722*	0.053*	-0.062*	-0.008
SPOR	0.456*	-0.02	0.167*	-0.096*
E1N	0.054	0.572*	0.213*	-0.039
E2N	-0.038	0.482*	0.114	0.03
E3N	-0.004	0.974*	-0.044*	0.049
E4N	0.007	0.930*	-0.027	-0.01
E5N	-0.022	0.303*	0.170*	-0.013
E6N	-0.066	0.144	0.029	-0.051
F8N	0.003	0.012	0.199*	0.248*
F9N	0.061	0.289*	0.194*	-0.013
F11N	0.034	0.224*	0.474*	0.054
F12N	-0.014	-0.086	0	0.700*
F13N	0.026	-0.039	0.190*	0.05
F14N	0.025	0.104*	0.239*	0.519*
F15N	-0.002	0.002	0.444*	0.106
F16N	0.045	-0.141*	-0.022	0.136*
F17N	-0.067	0.043	0.479*	0.135*
A5N	-0.054	0.280*	0.355*	-0.038
A6N	0.048	0.005	0.560*	-0.123*
A8N	0.025	0.235*	0.337*	0.003
C2N	0.032	0.188*	0.251*	-0.058
C4N	-0.004	0.122*	-0.036	-0.139*
C5N	0.059	0.140*	0.347*	0.09
C6N	-0.021	-0.026	0.607*	0.137*

Table S6. Exploratory factor analysis showing factor loadings for EAGL-short scale items across four factors. Asterisks (*) indicate statistically significant loadings. Variables represent different dimensions of genetic knowledge and attitudes, with loadings ranging from -0.166 to 0.974.

Table S7. Eigenvalues and Variance Explained for Exploratory Factor Analysis of the EAGL-long			
Factor	Eigenvalue	% of Variance	Cumulative %
Factor 1	3.744	12.48	12.50
Factor 2	2.268	7.56	20.0
Factor 3	1.096	3.65	23.70
Factor 4	0.615	2.05	25.70
Factor 5	1.099	3.66	29.4
Factor 6	0.984	3.28	32.7
Factor 7	1.044	3.48	36.2
Factor 8	1.257	4.19	40.4
Factor 9	0.680	2.27	42.60

Table S7. This table presents the eigenvalues and variance explained for each factor identified through Exploratory Factor Analysis of the EAGL-long measure. Each row represents a factor, ordered from highest to lowest eigenvalue. The first column presents the eigenvalues, or how much variance each factor explains. Those above the Kaiser criterion (eigenvalues > 1) are considered to be significantly meaningful. The second column presents this another way, through the percentage of total variance in the data that each factor accounts for individually. The third column, cumulative percentage, shows the total variance explained when you include each factor with the previous factors listed. All nine factors together explain 42.60% of the total variance in the data.

Table S8. Descriptive Statistics for Subjective Knowledge Items				
	Mean	SD	Skewness	Kurtosis
Genetic	6.09	0.99	-1.13	1.59
Chromosome	5.76	1.12	-0.86	0.99
Susceptibility	5.73	1.29	-1.25	1.87
Mutation	5.89	1.15	-1.24	2.07
Variation	5.38	1.46	-0.94	0.61
Heredity	6.1	1.04	-1.45	3.13
Sporadic	4.92	1.68	-0.66	-0.31
Overall	5.69	0.95	-0.69	0.48

Table S8. This table presents the descriptive statistics for the subjective knowledge items in the EAGL-short. Items were presented on a Likert scale, ranging from not at all familiar (0) to completely familiar (7). Mean scores, standard deviation, skewness, and kurtosis are presented.

Table S9. Frequency distribution for Knowledge Comprehension and Conceptual Knowledge items in CFA Sample (EAGL3, n = 1001)						
Factor	Item	1 (correct)	%	0 (incorrect)	%	n
Knowledge Comprehension	D1 (E1)	912	91.11	89	8.99	1001
	D2 (E2)	979	97.80	22	2.20	1001
	D3 (E3)	868	86.71	133	13.29	1001
	D4 (E4)	835	83.42	166	16.58	1001
Conceptual Knowledge	E1_1 (F11)	735	73.43	266	26.57	1001
	E1_2 (F14)	760	75.92	241	24.08	1001
	E1_3 (F15)	718	71.73	283	28.27	1001
	E1_4 (F17)	807	80.82	194	19.38	1001
	E2_1 (A6)	745	74.43	256	25.57	1001
	E2_2 (C6)	758	75.72	243	24.28	1001

Table S9. This table presents the frequency distributions for knowledge comprehensions and conceptual knowledge items within the sample used for CFA (EAGL3 sample, n = 1001). EAGL1 and EAGL2 were utilized for exploratory factor analysis (EFA) to identify the underlying factor structure, and EAGL3 for CFA to confirm the identified structure. This approach ensures a more rigorous validation of the factor model. Frequencies and percentages show the number and proportion of participants who answered each item correctly (1) or incorrectly (0). Items D1-D4 measure Knowledge Comprehension, while items E1_1-E2_2 measure Conceptual Knowledge as identified in exploratory factor analysis.

Table S10. Average Scores Per Subscale for EAGL-long and EAGL-short				
Instrument	Subscale	Mean	SD	Total Possible Score
EAGL-long	Subjective Knowledge	5.57	0.97	8
	Applied Knowledge	6.96	1.12	8
	Situational Knowledge	5.57	1.15	7
	Knowledge Comprehension	5.41	0.91	6
	Objective Knowledge	14.49	1.86	17
	Total Overall Score	38	3.86	46
EAGL-short	Subjective Knowledge	5.71	0.95	7
	Knowledge Comprehension	3.68	0.70	4
	Conceptual Knowledge	4.5	1.40	6
	Total Overall Score	13.89	2.01	17

Table S10. This table presents the average scores per subscale for both the EAGL-long and EAGL-short measures. The mean and standard deviation are presented for each subscale, along with for the overall score within each measure. The total possible scores are also presented.

Table S11. Adjusted Mean Estimates for Each Subscale in EAGL-short				
Subjective Knowledge	Variable	Value	Mean	Std. Err.
	Age	26-39	5.870501	0.048366
		40-49	5.956	0.055027
		50-59	5.886153	0.058928
		60-69	5.756377	0.069122
		70+	5.971	0.113743
		18-25	5.789449	0.06097
	Education	High school diploma or equivalent	5.725129	0.03996
		Associate degree	5.799588	0.054729
		Bachelor’s degree	5.836788	0.046494
		Professional degree	5.947521	0.062135
		Doctorate degree	5.953307	0.121663
		Less than High School	5.967149	0.143305
	Metro Status	Metro	5.821216	0.040745
		Nonmetro	5.921945	0.062807
	Connection to Autism	Yes	5.966647	0.056015
		No	5.776513	0.043638
Knowledge Comprehension	Age	26-39	3.643372	0.039485
		40-49	3.69233	0.043838
		50-59	3.732049	0.046419
		60-69	3.760242	0.053693
		70+	3.740388	0.085899
		18-25	3.65797	0.048235
	Education	High school diploma or equivalent	3.713923	0.031255
		Associate degree	3.713519	0.045621
		Bachelor’s degree	3.774669	0.039513
		Professional degree	3.552954	0.05662
		Doctorate degree	3.819741	0.117567
		Less than High School	3.651545	0.127593
	Metro Status	Metro	3.697003	0.034415
		Nonmetro	3.71178	0.049059
	Connection to Autism	Yes	3.720518	0.059353
		No	3.688265	0.034312
Conceptual Knowledge	Age	26-39	4.408883	0.087295
		40-49	4.322871	0.094327
		50-59	4.345177	0.100913
		60-69	4.303135	0.112935
		70+	4.442354	0.174413
		18-25	4.448759	0.102803
	Education	High school diploma or equivalent	4.432778	0.065618
		Associate degree	4.440231	0.104713
		Bachelor’s degree	4.656137	0.097307
		Professional degree	4.333828	0.156439
		Doctorate degree	4.630176	0.242011
		Less than High School	3.77803	0.333141
	Metro Status	Metro	4.501379	0.06269
		Nonmetro	4.255681	0.14664
	Connection to Autism	Yes	4.38015	0.095447
		No	4.37691	0.081367

Table S11. Supplementary table 11 presents the adjusted mean estimates for each demographic group across three subscales of the EAGL-short measure: subjective knowledge, knowledge

comprehension, and conceptual knowledge. Columns from left to right present the variables of interest, values of interests, adjusted means and standard error. Adjusted mean estimates control for the influence of other demographic variables on the variable of interest. Higher adjusted means indicate higher average scores within each subscale for the respective demographic group.
