# Supplementary material for: Psychometric validation of the education and assessment of genetic literacy or the EAGL measure
Source: HGG Adv. 2026 Jul 8;7(4):100651. doi: 10.1016/j.xhgg.2026.100651 (PMC13396853; doi:10.1016/j.xhgg.2026.100651)
Supplement: Document S2. Article plus supplemental information [file mmc2.pdf]

HGG
Advances

ARTICLE

Psychometric validation of the education and assessment of genetic literacy or the EAGL measure

Lily S. Barna,[1,2] Yi Liao,[3] Michael R. Wierzbicki,[4] Gabriela M. Ramírez-Renta,[1,5] Kimberly A. Kaphingst,[3] and Chris Gunter[1,6,7,*]

Summary

Genetic literacy is an integral measure for examining society's interaction with genetics, but widely used "genetic literacy" measures lack both knowledge comprehension measures and psychometric validation. To address these issues, we validated the education and assessment of genetic literacy (EAGL) measure in 2,708 US English-speaking general public participants, using both exploratory and confirmatory factor analysis. In addition to standard subjective and objective knowledge subscales, our measure's distinct knowledge comprehension subscale focuses on autism as an example of a complex condition. Regression analyses showed a statistically significant interaction when looking at education and personal connection to autism in relation to knowledge comprehension ($F = 3.68$, $p = 0.003$). Separately, those in our sample with a connection to autism scored higher only on the subjective knowledge section ($F = 19.52$, $p < 0.001$), concurring with previous demonstrations of a subjective-objective knowledge gap in science literacy. We then explored geographic location as one potential factor in genetic literacy and found that, contrary to expectations, metropolitan vs. non-metropolitan status had no significant main effects on literacy levels. After the validation process, we have two multi-domain measures that accurately capture the construct of genetic literacy and are available for wide use: the 46-question EAGL-long, which has previously been tested with thousands of participants, and the validated 17-question EAGL-short. The instruments will allow researchers to accurately gauge the complex genetic literacy levels of any population they wish to survey, helping to create more targeted and productive genetic communication interventions and educational materials.

Introduction

As accessibility to individual genomic information increases and raises corresponding healthcare implications, there is an equivalent need to increase societal and individual genetic literacy. Genetic knowledge alone is insufficient; we must aim for genetic literacy, defined as "the sufficient knowledge and understanding of genetic principles for individuals to make decisions that sustain personal well-being and effective participation in social decisions on genetic issues."[1,2] Given its importance, it is crucial to have a validated, effective, and reliable measure to measure genetic literacy levels. We therefore set out to adapt and modernize previously existing measures to address these constraints.

Genetic literacy exists in a larger expanse of literacies and understandings that may interconnect in a variety of ways. Health literacy, defined as "the ability of an individual to obtain and translate knowledge and information in order to maintain and improve health in a way that is appropriate to the individual and system contexts," has been researched individually and in connection with genetic literacy.[3] Numeracy, defined as "how facile people are with basic probability and mathematical concepts," has been widely associated with how people perceive health risks, linking it with health literacy.[4] Research shows those with lower health literacy and numeracy may struggle to process genetic information in both print and oral forms; however, studies using discriminant validity have confirmed genetic literacy represents a distinct construct.[5–8]

One difficulty for the field is that instruments attempting to capture genetic literacy vary widely, in fact capturing different subsets of genetic knowledge and application. The two most commonly measured subdomains are subjective knowledge (also called familiarity, or what someone thinks they know) and objective knowledge (also called knowledge, or what they actually know). Validated instruments such as the Rapid Estimate of Adult Literacy in Genetics (REAL-G)[9] and the Genetic Literacy and Comprehension (GLAC) measure assess familiarity with commonly found genetic terms.[10] The validated Genetic Knowledge Index (GKI) focuses solely on objective knowledge, asking true/false questions rooted in core genetic conceptual understanding.[11] Measures such as those used by Ishiyama et al.[12] investigate subjective knowledge, objective understanding of genetic concepts, and awareness of the benefits and risks present in regard to

[1]Engagement Methods Unit, Social and Behavioral Research Branch, National Human Genome Research Institute, Bethesda, MD 20892, USA; [2]Department of Communication Studies, Northwestern University, Evanston, IL 60208, USA; [3]Huntsman Cancer Institute and Department of Communication, University of Utah, Salt Lake City, UT 84112, USA; [4]Emmes Company, Rockville, MD 20850, USA; [5]University of Colorado Anschutz Medical School, Aurora, CO 13001, USA; [6]Office of the Director, National Human Genome Research Institute, Bethesda, MD 20892, USA
[7]Lead contact
*Correspondence: chris.gunter@nih.gov
https://doi.org/10.1016/j.xhgg.2026.100651.
Published by Elsevier Inc. on behalf of American Society of Human Genetics.
This is an open access article under the CC BY license (http://creativecommons.org/licenses/by/4.0/).

genomic studies. For objective knowledge, many genetic literacy measures (including ours) borrow from, update, and amend preexisting instruments such as Fitzgerald-Butt et al.,[13] updating and psychometrically evaluating the widely used genetic knowledge measure created by Jallinoja and Aro.[14] Finally, the International Genetics Literacy and Attitudes Survey (iGLAS-GK) measures genetic knowledge, heritability estimates, and attitudes toward various aspects of genetics use in education, in relation to the environment, in disease treatment, and more.[15]

A systematic review and survey to examine correlations and differences among six of the most commonly encountered instruments by Daly and Kaphingst[7] found two critical gaps in current genetic literacy measures. First, they noted a lack of subscales for knowledge comprehension (the ability to interpret and apply genetic information presented in context, for example, through infographics or scenarios) and/or applied knowledge (for example, using genetic terminology correctly in sentences). Of the 89 studies examined, only two articles examined knowledge comprehension as an aspect of genetic literacy; these use the Genetic Literacy Survey (GLS[2]), composed by the Social and Behavioral Research Branch (SBRB) of the National Human Genome Research Institute (NHGRI)[2,16] and later adapted by our unit into the Genetics and Autism Literacy Survey (GALS[1]). We then used GALS to examine genetic literacy in both the general population and in a large autism genetics research study, noting that general population levels in the US have increased slightly in the decade since the GLS.[1] We also showed that education level and confidence in one's own genetic knowledge were the largest contributors to genetic literacy components.[17] After the 2021 survey, we thoroughly reviewed our measure and ameliorated issues regarding language, content, and outdatedness of specific statements (see subjects and methods), generating the education and assessment of genetic literacy (EAGL) measure, specifically the 46-item EAGL-long.

Second, the review by Daly and Kaphingst[7] demonstrated a lack of psychometric evaluation and validation. Clarity is needed in many steps of measuring genetic literacy: from defining the term itself, to distinguishing between subjective and objective knowledge, and to capturing the critical component of knowledge comprehension. Therefore, this study aimed to validate the EAGL measure as a thorough and widely applicable instrument.

We conducted an iterative validation process in three phases, collected from December 2024 through February 2025. Following data collection, we performed exploratory factor analysis (EFA) and confirmatory factor analysis (CFA) to refine the instrument, resulting in a validated, three-factor version: the 17-item EAGL-short. Our results demonstrate that the EAGL-short effectively captures three core constructs of genetic literacy: subjective knowledge, knowledge comprehension, and conceptual knowledge/objective knowledge. These findings provide researchers and clinicians with a validated tool for measuring genetic literacy.

Subjects and methods

Sample

Ethics

All participants provided informed consent before participating in the survey and received compensation of $5.00. Participants were recruited via the online platform Prolific (see web resources), through which they were directed to an entirely online form via SurveyMonkey (see web resources). The average completion time ranged from 10 to 20 min, depending on the additional *ad hoc* questions included in the survey. The study was determined to be exempt from human subjects research by the National Institutes of Health Institutional Review Board (IRB002137/MOD008338).

Sample group characteristics

We collected data in three sequential survey waves between July 2024 and February 2025, using a sequential sampling strategy to maximize our statistical power and have samples for both EFA and CFA. The first two samples (n = 1,005 and n = 702) were combined for the EFA, while the third sample (n = 1,001) was reserved for CFA to validate the factor structure identified in the EFA. Summary demographic details for the combined sample can be found in Table 1, with more complete details for each run in Table S1.

The first sample (EAGL1, n = 1,005) was collected in two phases: 302 responses were collected on July 24, 2024, and 721 responses were collected between July 31 and August 4, 2024. The primary goal of the first sample was to ensure we could obtain high-quality data and explore how long data collection would take. This sample was designed to be nationally representative of the adult, English-speaking population in the US according to gender, age, and ethnicity. The nationally representative proportions are calculated from the US Census Bureau population group estimates from 2021.[18] After the removal of incomplete and invalid responses, 1,005 participants remained in the final dataset.

We found that 57.8% of the EAGL1 sample held a bachelor's degree or higher education, which exceeded national representation. Given our previous data demonstrating education as a moderating factor for genetic literacy overall,[17] the EAGL2 sample (n = 702) was collected on December 3 and 4, 2024, to oversample those with high school education and below.[19] We introduced attention check questions (designed to identify participants who are not reading questions carefully) into EAGL2 and -3 to help streamline the data cleaning process and ensure the validity of responses. After removing incomplete and invalid responses from the 714 collected, along with those who failed the attention check question, we were left with 702 responses, with this sample containing 22.4% of participants with a bachelor's degree or higher.

The EAGL3 sample (n = 1,001) was collected on February 19, 2025, and we designed it to match the combined previous two samples in educational distribution. A total of 1,001 remained after we cleaned the original 1,010 responses by removing incomplete and invalid responses, along with those who failed the attention check questions.

For regression analyses and other statistical tests, we utilized the combined dataset of all three samples to maximize statistical power. However, we had to remove four samples at this point because of invalid ZIP codes: one from EAGL1, two from EAGL2, and one

Table 1. Participant characteristics for combined sample ($N = 2{,}708$)

Variable	Category	Frequency	Percentage
Age	18–25	436	16.1%
	26–39	962	35.5%
	40–49	538	19.9%
	50–59	422	15.6%
	60–69	272	10.0%
	70+	78	2.9%
Education	kindergarten through 8th grade	5	0.2%
	some high school, no diploma	41	1.5%
	high school diploma or equivalent	1,095	40.4%
	associate degree	407	15.0%
	bachelor's degree	785	29.0%
	professional degree	311	11.5%
	doctorate degree	64	2.4%
Connection to autism	no	2,136	78.9%
	yes	572	21.1%
Metro status	metro	2,374	87.67%
	non-metro	330	12.19%
	invalid responses	4	0.15%

This table displays the demographic characteristics of the total study sample ($N = 2{,}708$). Age groups range from 18 to 70+ years. Education levels span from elementary through doctoral education. Connection to autism indicates whether participants reported any personal or familial connection to autism. Metro status categorizes participants' geographic location as metropolitan or non-metropolitan based on Federal Information Processing System (FIPS) codes for states and counties and Rural-Urban Continuum Codes (RUCC; see web resources).

from EAGL3; this produced $N = 2{,}704$ for all tests after the EFA/CFA. We examined the consistency of scores across all knowledge domains to ensure the appropriateness of combining the data. For confirmation, we ran a coefficient of variation (CV) analysis on the EAGL-long domains, which revealed excellent consistency across all knowledge domains, with CV values ranging from 0.29% for subjective knowledge to 1.35% for knowledge comprehension (Table S2). These exceedingly low values (all <1.5%) indicate minimal variation across samples, supporting our choice to combine the datasets for analysis.[20]

Measures

Genetic literacy

We measured genetic literacy using the EAGL measure, first developed by Abrams et al.[2,16] as the Genetic Literacy Survey (GLS) and later adapted by our laboratory as the Genetics and Autism Literacy Survey (GALS).[1] We then added two more subscales to test other aspects of genetic literacy: applied knowledge and situational knowledge. We swapped the term "abnormality" for "genome" in our survey to reflect our interests as the NHGRI while shifting away from the normal/abnormal binary that scholars have identified as problematic/ableist language.[21,22] Each iteration expanded the measure, including replacing *BRCA*-related material with an autism-related infographic adapted from Hoang et al.[23] and making amendments for language-preference issues, outdated research, and other content issues identified through our review. The full EAGL-long measure contains five subscales: subjective knowledge, applied knowledge, situational knowledge, knowledge comprehension, and objective knowledge (shown in Table S3 with correct answers given and described below).

Description of subscales

To assess subjective knowledge, or familiarity, participants rate their familiarity with eight common genetic terms: genetic, chromosome, susceptibility, mutation, variation, genome, heredity, and sporadic. Utilizing a seven-point Likert scale, answers ranged from not at all familiar (1) to completely familiar (7).

The applied knowledge section consists of eight corresponding multiple-choice questions, utilizing the same genetic terms from the subjective knowledge section in a sentence. Participants must answer multiple-choice questions in a Cloze-style technique, with only one correct choice per question.

We created the situational knowledge questions to reflect potential situations in which one needs genetic understanding. Participants answer six multiple-choice questions with subtopics including Mendelian inheritance, polygenic traits, genetic testing purposes, gene therapy, cloning, and sex-linked traits.

The knowledge comprehension (previously called "skills") section contains a pedigree with cups and balls demonstrating gene-environment interactions that can result in reaching the threshold for an autism diagnosis (adapted from Hoang et al.[23]), as a model of a complex condition. Participants are then asked six questions, scoring one point for every correct answer. The infographic is available for viewing at any point while answering the questions. Questions cover topics such as the purpose of genetic testing in autism, the impact of environmental and genetic factors, and the likelihood of autism diagnoses among family members.

The objective knowledge (previously called "knowledge") section consists of 17 true/false questions centered on multiple concepts within genetics. Participants answer "true" or "false" for each statement, with topics covering gene-environment interactions, heredity and condition carriers, mutations, and similar core genomic concepts. Correct responses earn one point, while incorrect responses receive zero points, resulting in a score range of 0–17.

Other measures

We measured objective numeracy using a three-item measure from Lipkus et al.[4] Participants answered three multiple-choice questions that centered on basic probability, turning a percentage into a proportion, and identifying risk magnitudes when presented as proportions.

Because our knowledge comprehension infographic and questions focus on autism, we asked the participants, "Are you or is anyone in your immediate family autistic?" The possible answers to "connection to autism" were yes or no.

Metropolitan or non-metropolitan distinctions were made via Federal Information Processing System (FIPS) codes for states and counties and Rural-Urban Continuum Codes (RUCC; see web resources). A RUCC of 1–3 delineates a metropolitan area, with 4–9 delineating a non-metropolitan county. The specific numbers indicate the level of adjacency to a metropolitan area, considering the population of the county and proximity to metro counties. As stated above, four participants had to be removed from regressions and statistical analyses because they provided invalid 5-digit ZIP codes.

Statistical analysis

Using R Statistical v.4.3.3 and v.4.4[24] and SAS (v.9.4), we performed data preparation with readr,[25] dplyr,[26] and tidyverse[27] and used reshape2[28] for data restructuring. We utilized the car package[29] and proc glm for analysis of variance (ANOVA), multcomp[30] for pairwise comparisons, and jtools[31] and proc glm/logistic for regressions. CFA path analysis was conducted with lavaan,[32] qgraph,[33] and MASS.[34] Data were visualized with ggplot2[35] and ggpubr,[36] and the subsequent analyses and visualizations were exported with writexl[37] and broom.[38] Missing data were handled by removing any participant with missing data on variables included in a given analysis.

We combined our first two samples, EAGL1 and EAGL2 (n = 1,707), for EFA using Mplus 8[39] statistical software. For factual questions encompassing applied knowledge, situational knowledge, and objective knowledge items, we calculated accuracy rates. Items with accuracy rates exceeding 90% were removed prior to EFA, as they lack sufficient variability to discriminate between participants and may not contribute meaningfully to identifying distinct factors of genetic literacy. Accuracy rates for all items in the final three-factor solution are presented in Table 2, with complete accuracy rates for all EAGL-long items available in the supplemental information. For EFA, we employed direct oblimin rotation, a method that permits correlations between items, and set a fixed number of factors.[40] The selection of items was based on Howard's[41] "40-30-20" guideline, requiring items to demonstrate a minimum primary factor loading of 0.40 while avoiding cross-loadings on other factors that exceed 0.30, except when the primary loading surpasses the secondary loading by at least 0.20.

We employed CFA to verify the factor structure discovered through EFA using EAGL3 (n = 1,001). Due to the binary nature of some items, the weighted least squares mean and variance adjusted (WLSMV) estimator was used. This method assumes that observed categorical responses reflect an underlying continuous normal distribution segmented by threshold values that govern the transformation of continuous scores into categorical outcomes, as opposed to treating the categorical responses as inherently normally distributed.[42] We used multiple indices to assess model fit: comparative fit index (CFI), root-mean-square error of approximation (RMSEA), and standardized root-mean-square residual (SRMR). A CFI > 0.95, an RMSEA < 0.06, and an SRMR < 0.08 all indicate good model fit.[43] Cronbach's α was utilized to measure the internal consistency for each subscale, with a threshold of 0.70 or higher.

We chose EFA and CFA, as opposed to item response theory (IRT), for several methodological reasons. Our theoretical framework posits that genetic literacy is a multidimensional construct composed of distinct but interrelated factors (subjective knowledge, objective knowledge, and knowledge comprehension), each representing a different aspect of how individuals understand and process genetic information. Given that genetic literacy is a construct with multiple dimensions, factor analysis is optimal for accounting for these qualities. Additionally, EFA and CFA are more appropriate for measures with varying question types, as our measure includes Likert scale, true/false, numeric answer, and multiple-choice questions. The larger sample size allowed us to explore the relationships between variables we observed, as opposed to IRT, which is more probabilistic.

We performed linear regressions to examine associations between our genetic literacy outcome measures and predictor variables (age, education, connection to autism, numeracy, and metro status). ANOVA was used to test the significance of effects and interactions in the regression models. Models included interaction terms between education and the other predictor variables to see if educational effects varied among groups. Sensitivity analyses were performed to assess the robustness of the results of the ANOVA models. Finally, we performed cumulative logit regression models with the same predictor values and Kruskal-Wallis tests for each predictor.

For the categorical variable of education, the variable "less than high school" serves as a reference level, with that group composed of all responses of "no schooling completed," "kindergarten through 8th grade," and "some high school, no diploma" (n = 46). We performed education and metro status analyses on the full dataset (N = 2,704), assessing model fit using F-statistics, p values, and R^2 values.

Results

Descriptive statistics

The final combined sample (N = 2,704) included US-based English-speaking participants across age groups, educational attainment groups, and metropolitan/non-metropolitan groups (see Tables 1 for a combined summary and S1 for more details). The largest age representation (35.5%) is 26–39, while the largest educational grouping was those with a high school diploma or equivalent (40.4% of the sample). 78.9% of participants reported no connection to autism either personally or in their family, while 21.1% reported having a connection. The majority (87.7%) of our participants resided in metropolitan areas.

Table 2. Factor loadings, communalities, and accuracy rates for EAGL-short (EFA)

	Variable	Factor 1	Factor 2	Factor 3	h^2	Accuracy rate	Question
Subjective knowledge	GENE	0.822[a]	0.010	−0.147	0.697	–	–
	CHRO	0.800[a]	−0.072	−0.002	0.645	–	–
	SUSC	0.721[a]	0.091	0.036	0.529	–	–
	MUTA	0.835[a]	0.030	−0.010	0.698	–	–
	VARI	0.634[a]	−0.058	0.140	0.425	–	–
	HERE	0.734[a]	0.038	−0.092	0.549	–	–
	SPOR	0.483[a]	0.006	0.083	0.240	–	–
Knowledge comprehension	D1 (E1)	0.057	0.586[a]	0.190	0.383	87.93%	Q: What is the purpose of genetic testing for autism? correct A: Genetic testing analyzes someone's DNA to find genetic variations related to autism.
	D2 (E2)	−0.051	0.482[a]	0.135	0.253	98.30%	Q: Please select the phrase that best completes the following statement: Genetic variations that could increase a person's chance of being autistic … correct A: Can be present in some siblings and not others.
	D3 (E3)	−0.027	0.959[a]	−0.006	0.920	94.20%	Q: What percentage of individuals who have genetic testing for autism are found to have a variation related to autism? correct A: 25%
	D4 (E4)	−0.005	0.930[a]	−0.022	0.865	92.85%	Q: What percentage of individuals who have genetic testing for autism will receive results with no genetic variations related to autism? correct A: 75%
Conceptual knowledge	E1_1 (F11)	0.032	0.245	0.483[a]	0.294	74.93%	Q: A gene is a cell. correct A: False
	E1_2 (F14)	−0.052	0.037	0.474[a]	0.229	74.75%	Q: Genes are bigger than chromosomes. correct A: False
	E1_3 (F15)	−0.011	0.016	0.481[a]	0.232	72.76%	Q: The genome can be changed by human intervention. correct A: True
	E1_4 (F17)	−0.083	0.054	0.533[a]	0.294	80.37%	Q: Environmental factors, such as UV radiation, can change our DNA sequence. correct A: True
	E2_1 (A6)	0.085	0.063	0.458[a]	0.221	73.52%	Q: Your genome is … correct A: Your entire and complete set of DNA
	E2_2 (C6)	−0.032	−0.007	0.654[a]	0.429	73.11%	Q: The term DNA stands for … correct A: Deoxyribonucleic acid

This table presents results from exploratory factor analysis (EFA) of the EAGL-short instrument. h^2 = communality, or the proportion of variance in a measured variable explained by the underlying factors (latent constructs).[40] Accuracy rates are provided for knowledge comprehension and conceptual knowledge items, indicating the percentage of participants who answered each question correctly. Each knowledge comprehension or conceptual knowledge item is listed as a position in EAGL-short vs. EAGL-long, e.g., E1_1 (F11) is item E1_1 in EAGL-short but was item F11 in EAGL-long.
[a]Primary loadings >0.40 for each variable.

EFA

We conducted EFA on the 46-item EAGL measure (Table S3), initially identifying nine factors centering on various subject and knowledge domains (Table S4; Figure S1D). We ran EFA analyses fixing the factor number at five (Table S5), four (Table S6), and three (Table 2) and followed the 40-30-20 guideline[41] for item selection. Heatmap representations of these four EFA analyses are in Figures S1A–S1D. We found three strong factors present throughout the measure, distilling the measure down to a three-factor solution with clear groupings: subjective knowledge, knowledge comprehension, and objective knowledge (Table 2). Items that did not load significantly onto any of the three factors (with loadings below 0.4) were identified for potential removal in the subsequent CFA phase, with the final validated survey becoming the EAGL-short. Eigenvalues and variance explained for all factors are presented in Table S7. We established a threshold of 0.4 as a baseline, with two items having loadings just under 0.4 but still retained due to the larger

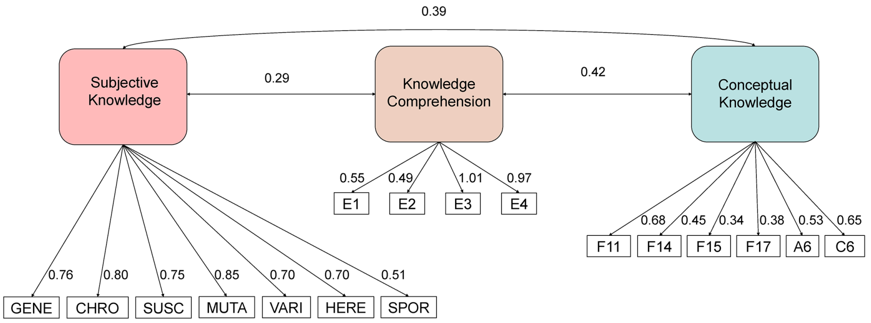

Figure 1. Confirmatory factor analysis path diagram for the EAGL-short (n = 1,001), estimated using the weighted least squares mean and variance adjusted estimator in lavaan
For the knowledge comprehension and conceptual knowledge items, the number in each box (e.g., "F11") corresponds to the item's original position in the EAGL-long. Latent factors (ovals) are connected to their observed indicator items (rectangles) by single-headed arrows; standardized factor loadings are shown alongside each path. Curved double-headed arrows between latent factors represent standardized factor correlations. Model fit indices: CFI = 0.996, RMSEA = 0.031 [0.025, 0.037], and SRMR = 0.080.

sample size and theoretical and practical relevance of the items.[44]

CFA

We performed CFA on the three-factor model identified through EFA, after removing items that failed to load significantly on any factor. We removed items A1–A5, A7, A8, C1–C5, E5, E6, F1–F10, F12, F13, and F16 (see Table S3 for specifics), as well as the term "genome," as each item either loaded on multiple factors or failed to load on any factor with statistical significance. The resulting CFA model consists of 17 genetic literacy questions, constituting the EAGL-short (Figure 1). Factor loadings, standard errors, and standardized loadings for all items are presented in Table 3.

The model demonstrated excellent fit across multiple indices. The CFI value (0.996 standard, 0.989 scaled) was above 0.95, indicating an excellent fit. The RMSEA of 0.031 [0.025, 0.037] was below the 0.05 threshold, also suggesting a good fit. The SRMR (0.080) is acceptable at the base threshold of ≤0.08. The chi-squared test of model fit (X^2 [116] = 115.426, p = 0.498) was non-significant, indicating no significant differences between the model data and observed data, suggestive of an exceptional fit.

Validated measure

After our factor analysis, the EAGL-short demonstrated strong psychometric properties among three validated factors: subjective knowledge, knowledge comprehension, and conceptual knowledge. Subjective knowledge (Table S8, 7-item Likert scale; M = 5.69, SD = 0.95, α = 0.87), knowledge comprehension (Table S9, 4-item multiple-choice and numeric answer; M = 3.68, SD = 0.97, α = 0.83), and conceptual knowledge (Table S9, 6-item multiple-choice and true/false; M = 4.54, SD = 1.40, α = 0.67) all showed acceptable to excellent internal consistency (see Tables S8 for detailed descriptive statistics of subjective knowledge items and S9 for frequency distributions of knowledge comprehension and conceptual knowledge items). Conceptual knowledge is an amalgamation of several questions from the applied knowledge, situational knowledge, and objective knowledge sections (A6, C6, F11, F14, F15, and F17). The shared factor of conceptual knowledge appears to center on fundamental genetics principles of heredity and genetic changes that do or do not lead to disease. A potential reason for the relatively low α-value of 0.67 is the heterogeneity of the question types in this section, with true/false, multiple-choice, and multiple-choice fill-in-the-blank-style questions.[45]

EAGL-long performance

The mean values for all five portions of the EAGL-long are presented in Table S10. Compared to previous administrations of our GLS, the results were slightly higher in this population.[1,17] The EAGL-long subscales showed variable internal consistency, with subjective knowledge demonstrating good reliability (α = 0.877), while other subscales showed lower α-values due to the heterogeneous nature of the items and the smaller number of questions per subscale. Consistency across samples was confirmed through CV analysis (Table S2), with all CV values below 1.5%.

Regression analysis

To examine how demographic variables predict the three constructs of genetic literacy, we performed regression analysis on the EAGL sample in its entirety, as well as on each of the three EAGL samples. The analysis centered on the EAGL-short subscales of subjective knowledge, knowledge comprehension, and conceptual knowledge. ANOVA revealed several statistically significant associations between demographic variables and EAGL-short subscales (Table 4). Complete adjusted means for each demographic group are available in Table S11.

Total numeracy score showed highly significant effects across all three subscales of EAGL-short (all $p < 0.001$), consistently demonstrating the strongest associations. In contrast, metropolitan (RUCC 1–3) vs. non-metropolitan (RUCC 4–9) status had no significant main effects on overall levels.

For subjective knowledge (scored 0–7), age (F = 3.06, p = 0.009) and connection to autism (F = 19.52, p < .001) showed significant effects in addition to numeracy. Those with a connection to autism scored higher (adjusted M = 5.97; Table S11) than those without (M = 5.78), though the effect size was small (Cohen's d = 0.19), indicating limited practical significance.

For knowledge comprehension (scored 0–4), numeracy was the only significant main effect predictor (F = 23.98,

Table 3. Factor loadings for EAGL-short (confirmatory factor analysis)

Factor	Indicator	Factor loading	Standard error	Z value	*p* value	Standardized factor loading
Subjective knowledge	genetic	1.000	–	–	–	0.759
	chromosome	1.184	0.040	29.594	<0.001	0.798
	susceptibility	1.279	0.049	26.238	<0.001	0.749
	mutation	1.295	0.045	28.587	<0.001	0.848
	variation	1.353	0.060	22.625	<0.001	0.699
	heredity	0.967	0.037	26.381	<0.001	0.701
	sporadic	1.133	0.073	15.481	<0.001	0.508
Knowledge comprehension	D1 (E1)	1.000	–	–	–	0.548
	D2 (E2)	0.898	0.196	4.592	<0.001	0.493
	D3 (E3)	1.841	0.212	8.691	<0.001	1.009
	D4 (E4)	1.772	0.200	8.867	<0.001	0.972
Conceptual knowledge	E1_1 (F11)	1.000	–	–	–	0.678
	E1_2 (F14)	0.666	0.102	6.561	<0.001	0.451
	E1_3 (F15)	0.496	0.094	5.280	<0.001	0.336
	E1_4 (F17)	0.556	0.098	5.699	<0.001	0.377
	E2_1 (A6)	0.788	0.106	7.447	<0.001	0.534
	E2_2 (C6)	0.951	0.112	8.524	<0.001	0.645

This table presents the findings from the confirmatory factor analysis (CFA) for the EAGL-short measure. Factor loadings fixed to 1.000 represent reference indicators for model identification. Standardized loadings >0.5 are considered adequate, and those >0.7 are excellent. Values <0.5 may be acceptable within the context if they contribute to the overall model fit. Each knowledge comprehension or conceptual knowledge item is listed as a position in EAGL-short vs. EAGL-long, e.g., E1_1 (F11) is item E1_1 in EAGL-short but was item F11 in EAGL-long.

$p < .001$). Neither connection to autism (F = 1.73, p = 0.188, Cohen's d = 0.04) nor any other demographic variable showed a significant main effect. We did observe a statistically significant interaction between education and connection to autism (F = 3.68, $p = 0.003$).

For conceptual knowledge (scored 0–6), both numeracy (F = 16.78, $p < 0.001$) and education (F = 4.04, $p = 0.001$) showed significant main effects. Those with bachelor's degrees (adjusted M = 4.66) and doctoral degrees (M = 4.63) scored notably higher than those with less than high school education (M = 3.78). A statistically significant interaction between education and metro status was also observed (F = 2.30, $p = 0.043$).

Discussion

This study reports the psychometric evaluation and validation of the EAGL measure in three robust US population samples. The analyses indicate that the EAGL-short instrument is sound and captures unique aspects of genetic literacy, a multidimensional construct that by definition should include a measurement of comprehension as opposed to only subjective or objective knowledge.

The exploratory and confirmatory factor analyses ultimately illustrate a highly factorial construct in the EAGL-long. The longer version is still composed of individually validated components and may serve in a variety of settings, including academic pre- and post-tests or genetic testing population surveys, while the shorter version may be more streamlined for clinical or exit-survey settings. The psychometrically validated three-factor structure in EAGL-short, which highlights subjective knowledge, knowledge comprehension, and conceptual knowledge, aligns with the previous dimensions (familiarity, knowledge, and skills) identified in both Little et al.[1] and Abrams et al.[2] The psychometric validation process identified several items among the five subscales in EAGL-long that either loaded on unique factor domains or did not discriminately load on any factor domain, thereby not measuring any specific aspect of genetic literacy. The EAGL-short condensed the more varied EAGL-long into a tight-knit body of effective questions.

Findings from a validation study of the EAGL predecessor showcase via discriminant validity analysis that genetic literacy is a distinct construct, separate from both health literacy and numeracy.[8] While it is clear these constructs coexist and likely relate to one another, genetic literacy uniquely homes in on the processing of genetic health information. Liao et al.[8] emphasize the need to examine these constructs both in relation to one another and separately to glean their truest effects on genetically related health decision-making.

The knowledge comprehension factor represents an integral and underexplored component of genetic literacy. With many previous measures focusing solely on subjective

Table 4. ANOVA statistics

Variable	Type II SS	Mean square	F value	Pr > F
**Subjective knowledge**				
Age	13.24	2.65	3.06	0.009
Total numeracy	50.27	25.14	29.08	<0.001
Education	9.43	1.89	2.18	0.053
Metro status	2.42	2.42	2.80	0.094
Connection to autism	16.87	16.87	19.52	<0.001
**Knowledge comprehension**				
Age	4.72	0.94	1.99	0.078
Total numeracy	22.77	11.39	23.98	<0.001
Education	4.34	0.87	1.83	0.104
Metro status	0.02	0.02	0.05	0.825
Connection to autism	0.82	0.82	1.73	0.188
Education * connection to autism	8.74	1.75	3.68	0.003
**Conceptual knowledge**				
Age	7.18	1.44	0.75	0.590
Total numeracy	64.65	32.33	16.78	<0.001
Education	38.91	7.78	4.04	0.001
Metro status	0.13	0.13	0.07	0.794
Connection to autism	0.01	0.01	0.01	0.942
Education * metro status	22.16	4.43	2.30	0.043

This table presents the analysis of variance (ANOVA) results for the EAGL-short subscales: subjective knowledge, knowledge comprehension, and conceptual knowledge. ANOVA tests for statistically significant differences between demographic groups on each subscale. Columns present the type II sum of squares (SS), representing the amount of variance explained by each variable; mean square, the sum of squares divided by degrees of freedom (average variance); F value, the test statistic comparing variance between groups to variance within groups; and *p* value, the probability the result occurred by chance (values <0.05 typically indicate statistical significance). Some models include interaction terms, which test if the effect of one variable depends on another variable. Note that there is no education * metro status interaction in subjective knowledge due to the model selection procedure. We ran the model with all two-way interactions with education, then re-ran it while keeping only the significant interactions from the first model.

and/or objective knowledge, the EAGL directly measures individuals' ability to synthesize and apply given genetic information. The strong participant scores throughout the section indicate that with cohesive and clear communication, synthesizing genetic information can be an equitable and universal accomplishment. Specifically using an example of the complex-condition model in autism showcases how individuals can understand complex scientific ideas, such as gene-environment interactions, effectively. Further research should investigate the longevity of their understanding of the information they synthesized in the knowledge comprehension section.

The overall participant scores in this study were higher than those of the previous measure iterations.[1,17] This may indicate higher genetic literacy levels overall consistent with Little et al.[1] finding that US-based genetic literacy levels rose in the general population over time; yet, they indicated continued need for improvement. It could also be due to increased public exposure to genetic information, as our previous data collection occurred in 2021, and genomics has only further made it to the forefront of mass media since then. Or, it could reflect qualities of the users of the Prolific platform compared to our previous public panels.

The findings that those with a personal or familial connection to autism scored higher on subjective knowledge, but not knowledge comprehension or conceptual knowledge, provide evidence for the subjective-objective knowledge gap present in health literacy broadly. The subjective-objective knowledge gap posits that the more people believe they understand the science, the more confident they may feel in their understanding of it.[46,47] Those with connections to autism may hold stronger feelings toward genetics, be it positive or negative, thereby finding themselves more familiar with genetic terms, even if they do not actually know other aspects of genetics.[46] Another potential reason for higher subjective knowledge is more exposure to genetic terms in their daily lives. Interactions with genetic counselors and genetic testing, increasingly common components of receiving an autism diagnosis, may contribute to increased familiarity with genetic terms.

The statistically significant interaction between education level and connection to autism in relation to

knowledge comprehension suggests that one's educational attainment may moderate how their personal connections relate to their genetic literacy levels. Those with higher educational attainment may have additional skills to interpret/understand genetic information that they have encountered through their personal or familial autism connection, whereas those with less education may have fewer skills through which their personal experiences can translate into higher genetic literacy. Another potential explanation is that those with higher educational levels may seek out more genetic information related to their autism connection and may have stronger comprehension skills overall regarding complex genetic information.

There were no main effects of metropolitan status on any of the three genetic literacy subscales, suggesting that geographic location measured as metro/non-metro alone does not predict genetic literacy levels. We did observe a statistically significant interaction between education and metro status for conceptual knowledge ($F = 2.30$, $p = 0.043$), though the specific pattern of this interaction requires further investigation to interpret.

While we hypothesized that those in metropolitan areas would have higher genetic literacy due to greater exposure to genetic concepts through healthcare services, testing opportunities, genetic counselors, and research institutions, our data did not support this hypothesis. The lack of main effects suggests that deficits in genetic literacy may be more universal than geography specific. However, the observed interaction may indicate that the relationship between education and conceptual knowledge differs between metropolitan and non-metropolitan contexts, potentially reflecting differences in educational quality, curriculum emphasis, or access to genetics-related healthcare services. Further research should investigate both the nature of this interaction and potential differences in genetic training among healthcare providers across geographic areas.

Through these validation efforts, the EAGL is ready for widespread use in a variety of social populations and settings. Still, further research could help establish its full efficacy in multiple social groups and languages. There is additional room for streamlining, and reorganization of the survey questions may also be a potential avenue of exploration, helping to further explicate the mediation and moderation of subjective knowledge, knowledge comprehension, and conceptual knowledge on one another. The findings that genetic literacy concepts are accessible across educational, age, and geographical groups have important implications for provider education, patient education, mass communication, genetic counseling, public health initiatives, and other groups that interact with genetic information. For those interested in the original scale and domain structure, the full EAGL-long remains available for use as a comprehensive coverage of genetic literacy, though researchers should be aware of the psychometric limitations of individual subscales.

The validation of a genetic literacy measure that captures subjective knowledge, objective knowledge, and knowledge comprehension has implications for both the continued field of genetic literacy research and the public as genetics continues to grow in clinical utilization. Understanding individuals' genetic literacy levels can inform providers about effective communication techniques and educational interventions. Psychometric validation allows researchers to accurately gauge the complex genetic literacy levels of any population they wish to survey, helping to create more targeted and productive genetic communication interventions and educational materials.

Conclusion

This study ultimately validates the EAGL measure as a psychometrically sound measure of genetic literacy. The analyses showcase a robust and multi-factorial long-form version that touches on many domains of genetic knowledge and literacy and a streamlined shortened version that encompasses three key factors of genetic literacy: subjective knowledge, knowledge comprehension, and conceptual knowledge. These factors are recognized as core constructs of genetic literacy, further solidifying EAGL as a measure suitable for widespread use. Knowledge comprehension, or skills, has been identified as a unique and valid construct, something absent from many previous genetic literacy measures but worth utilizing in future studies and interventions. With so much discrepancy among current genetic literacy definitions, measures, and implications, creating and identifying psychometrically sound instruments is of high importance.

While previous literature asserts a continued effect of education on genetic literacy levels, the results of this survey indicate an overall increase in score regardless of educational level, along with a non-significant difference in score among educational levels. The additional non-significant difference in score among those in metro and non-metro areas indicates that genetic literacy issues pervade educational and geographical strata. Genetic literacy needs to be evaluated and improved across the board to help empower patients and providers alike and increase informed decision-making. The validated EAGL measures, including the knowledge comprehension subscales, can be valuable tools for identifying current genetic literacy levels and gaps that need addressing.

Limitations

While this study provides robust validation of the EAGL measures, several limitations should be acknowledged. Utilizing an online survey recruitment platform may lead to selection bias and exclusion of those without access to technology.[48,49] The self-reporting of subjective knowledge may lead to implicit biases, with some overestimating or underestimating their own familiarity levels.[50] The use of autism as our example of the complex conditions model may introduce condition-specific biases, with those who have opinions on or prior

experience with autism. Researchers can address these limitations in future studies by surveying in-person or over the phone to reach participants without internet capabilities, introducing a more objective measurement of term recognition, or selecting a different example to illustrate the complex condition.[2] Additionally, this validation was run with only US participants who speak English as their first language. Running the validation in additional languages and locations could help increase the spread of the measure, ensuring it correctly captures genetic literacy across places and languages.

Data and code availability

The raw data utilized in this study were derived from a larger research project. The dataset will be publicly available after the initial articles reporting on the collected data are published. Summary statistics are published in Little et al.[1] Until then, the dataset can be accessed by contacting the corresponding author (chris.gunter@nih.gov) upon reasonable request.

Acknowledgments

This project was funded by a SPARK Research Match grant (RM0149) and the National Human Genome Research Institute Intramural Research Program (HG200410-01). The contributions of the NIH authors are considered works of the US government. The findings and conclusions presented in this paper are those of the authors and do not necessarily reflect the views of the NIH or the US Department of Health and Human Services. We thank Laura Koehly, Susan Persky, and the EMU and HCBU labs at NHGRI for suggestions throughout.

Author contributions

Conceptualization and experimental design, L.S.B., Y.L., G.M.R.-R., K.A.K., and C.G.; data analysis, L.S.B., Y.L., M.R.W., K.A.K., and C.G.; data curation, L.S.B., Y.L., G.M.R.-R., and M.R.W.; writing – original draft, L.S.B. and C.G.; writing – review & editing, L.S.B., Y.L., M.R.W., K.A.K., and C.G. All authors have reviewed and approved the final version of the manuscript.

Declaration of interests

The authors declare no competing interests.

Declaration of generative AI and AI-assisted technologies in the writing process

During the preparation of this work, the authors used Claude v.4.1 Opus in order to check grammar and generate visualizations. After using this tool/service, the authors reviewed and edited the content as needed and take full responsibility for the content of the publication.

Supplemental information

Supplemental information can be found online at https://doi.org/10.1016/j.xhgg.2026.100651.

Web resources

Prolific, https://www.prolific.com

SurveyMonkey, https://www.surveymonkey.com

USDA Rural-Urban Continuum Codes (RUCC), https://www.ers.usda.gov/data-products/rural-urban-continuum-codes/documentation

Received: March 21, 2026
Accepted: July 2, 2026

References

1. Little, I.D., Koehly, L.M., and Gunter, C. (2022). Understanding changes in genetic literacy over time and in genetic research participants. Am. J. Hum. Genet. *109*, 2141–2151. https://doi.org/10.1016/j.ajhg.2022.11.005.
2. Abrams, L.R., McBride, C.M., Hooker, G.W., Cappella, J.N., and Koehly, L.M. (2015). The many facets of genetic literacy: Assessing the scalability of multiple measures for broad use in survey research. PLoS One *10*, e0141532. https://doi.org/10.1371/journal.pone.0141532.
3. Liu, C., Wang, D., Liu, C., Jiang, J., Wang, X., Chen, H., Ju, X., and Zhang, X. (2020). What is the meaning of health literacy? A systematic review and qualitative synthesis. Fam. Med. Commun. Health *8*, e000351. https://doi.org/10.1136/fmch-2020-000351.
4. Lipkus, I.M., Samsa, G., and Rimer, B.K. (2001). General Performance on a Numeracy Scale among Highly Educated Samples. Med. Decis. Mak. *21*, 37–44. https://doi.org/10.1177/0272989X0102100105.
5. Lea, D.H., Kaphingst, K.A., Bowen, D., Lipkus, I., and Hadley, D.W. (2011). Communicating genetic and genomic information: health literacy and numeracy considerations. Public Health Genom. *14*, 279–289. https://doi.org/10.1159/000294191.
6. Kaphingst, K.A., Blanchard, M., Milam, L., Pokharel, M., Elrick, A., and Goodman, M.S. (2016). Relationships Between Health Literacy and Genomics-Related Knowledge, Self-Efficacy, Perceived Importance, and Communication in a Medically Underserved Population. J. Health Commun. *21*, 58–68. https://doi.org/10.1080/10810730.2016.1144661.
7. Daly, B.M., and Kaphingst, K.A. (2023). Variability in conceptualizations and measurement of genetic literacy. PEC Innov. *2*, 100147. https://doi.org/10.1016/j.pecinn.2023.100147.
8. Liao, Y., Wei, W., Barna, L.S., Gunter, C., and Kaphingst, K.A. (2026). Genetic literacy scale: Construct and discriminant validity and population differences. Patient Educ. Couns. *142*, 109390. https://doi.org/10.1016/j.pec.2025.109390.
9. Erby, L.H., Roter, D., Larson, S., and Cho, J. (2008). The rapid estimate of adult literacy in genetics (REAL-G): a means to assess literacy deficits in the context of genetics. Am. J. Med. Genet A *146*, 174–181. https://doi.org/10.1002/ajmg.a.32068.
10. Hooker, G.W., Peay, H., Erby, L., Bayless, T., Biesecker, B.B., and Roter, D.L. (2014). Genetic literacy and patient perceptions of IBD testing utility and disease control: a randomized vignette study of genetic testing. Inflamm. Bowel Dis. *20*, 901–908. https://doi.org/10.1097/MIB.0000000000000021.
11. Furr, L.A., and Kelly, S.E. (1999). The Genetic Knowledge Index: Developing a Standard Measure of Genetic Knowledge. Genet. Test. *3*, 193–199. https://doi.org/10.1089/gte.1999.3.193.

12. Ishiyama, I., Nagai, A., Muto, K., Tamakoshi, A., Kokado, M., Mimura, K., Tanzawa, T., and Yamagata, Z. (2008). Relationship between public attitudes toward genomic studies related to medicine and their level of genomic literacy in Japan. Am. J. Med. Genet. *146A*, 1696–1706. https://doi.org/10.1002/ajmg.a.32322.
13. Fitzgerald-Butt, S.M., Bodine, A., Fry, K.M., Ash, J., Zaidi, A.N., Garg, V., Gerhardt, C.A., and McBride, K.L. (2016). Measuring genetic knowledge: a brief survey instrument for adolescents and adults. Clin. Genet. *89*, 235–243. https://doi.org/10.1111/cge.12618.
14. Jallinoja, P., and Aro, A.R. (1999). Knowledge about genes and heredity among Finns. New Genet. Soc. *18*, 101–110. https://doi.org/10.1080/14636779908656892.
15. Chapman, R., Likhanov, M., Selita, F., and Zakharov, I. (2017). Genetic Literacy And Attitudes Survey (Iglas): International Population-Wide Assessment Instrument. European Proc. Soc. Behav. Sci. EpSBS *33*, 45–66. https://doi.org/10.15405/epsbs.2017.12.6.
16. Abrams, L.R., Koehly, L.M., Hooker, G.W., Paquin, R.S., Capella, J.N., and McBride, C.M. (2016). Media Exposure and Genetic Literacy Skills to Evaluate Angelina Jolie's Decision for Prophylactic Mastectomy. Public Health Genom. *19*, 282–289. https://doi.org/10.1159/000447944.
17. Ramírez Renta, G.M., Little, I.D., Koehly, L.M., Hilliard, A.J., Foor, K.L., Butts, J., Lundeen, J., and Gunter, C. (2025). Interaction of identity and beliefs with genetic literacy. Am. J. Hum. Genet. *113*, 16–28. https://doi.org/10.1016/j.ajhg.2025.11.014.
18. U.S. Census Bureau Explore Census Data.
19. Haga, S.B., Barry, W.T., Mills, R., Ginsburg, G.S., Svetkey, L., Sullivan, J., and Willard, H.F. (2013). Public knowledge of and attitudes toward genetics and genetic testing. Genet. Test. Mol. Biomark. *17*, 327–335. https://doi.org/10.1089/gtmb.2012.0350.
20. Reed, G.F., Lynn, F., and Meade, B.D. (2002). Use of coefficient of variation in assessing variability of quantitative assays. Clin. Diagn. Lab. Immunol. *9*, 1235–1239. https://doi.org/10.1128/cdli.9.6.1235-1239.2002.
21. Calder-Dawe, O., Witten, K., and Carroll, P. (2020). Being the body in question: young people's accounts of everyday ableism, visibility and disability. Disabil. Soc. *35*, 132–155. https://doi.org/10.1080/09687599.2019.1621742.
22. Bottema-Beutel, K., Kapp, S.K., Lester, J.N., Sasson, N.J., and Hand, B.N. (2021). Avoiding Ableist Language: Suggestions for Autism Researchers. Autism Adulthood *3*, 18–29. https://doi.org/10.1089/aut.2020.0014.
23. Hoang, N., Cytrynbaum, C., and Scherer, S.W. (2018). Communicating complex genomic information: A counselling approach derived from research experience with Autism Spectrum Disorder. Patient Educ. Couns. *101*, 352–361. https://doi.org/10.1016/j.pec.2017.07.029.
24. R Core Team. (2024). R: A Language and Environment for Statistical Computing (R Foundation for Statistical Computing).
25. Wickham, H., Hester, J., and Bryan, J. (2024). Readr: Read Rectangular Text Data. R package version 2.15.
26. Wickham, H., François, R., Henry, L., Müller, K., and Vaughan, D. (2023). Dplyr: A Grammar of Data Manipulation (version 1.1.2).
27. Wickham, H., Averick, M., Bryan, J., Chang, W., McGowan, L., François, R., Grolemund, G., Hayes, A., Henry, L., Hester, J., et al. (2019). Welcome to the tidyverse. J. Open Source Softw. *4*, 1686. https://doi.org/10.21105/joss.01686.
28. Wickham, H. (2007). Reshaping Data with the reshape Package. J. Stat. Softw. *21*, 1–20. https://doi.org/10.18637/jss.v021.i12.
29. Fox, J., and Weisberg, S. (2019). An R Companion to Applied Regression, Third Edition (Sage).
30. Hothorn, T., Bretz, F., and Westfall, P. (2008). Simultaneous Inference in General Parametric Models. Biom. J. *50*, 346–363. https://doi.org/10.1002/bimj.200810425.
31. Long, J.A. (2022). Jtools: Analysis and Presentation of Social Scientific Data.
32. Rosseel, Y. (2012). lavaan: An R Package for Structural Equation Modeling. J. Stat. Softw. *48*, 1–36. https://doi.org/10.18637/jss.v048.i02.
33. Epskamp, S., Cramer, A.O.J., Waldorp, L.J., Schmittmann, V.D., and Borsboom, D. (2012). qgraph: Network Visualizations of Relationships in Psychometric Data. J. Stat. Softw. *48*, 1–18. https://doi.org/10.18637/jss.v048.i04.
34. Venables, W.N., and Ripley, B.D. (2002). Modern Applied Statistics with S, Fourth Edition (Springer).
35. Wickham, H. (2016). ggplot2: Elegant Graphics for Data Analysis (Springer-Verlag).
36. Kassambara A. Ggpubr: 'ggplot2' Based Publication Ready Plots. R package 0.6. 2023.
37. Ooms, J. (2025). Writexl: Export Data Frames to Excel 'xlsx' Format. R package version 1.5. 2025.
38. Robinson, D., Hayes, A., and Couch, S. (2024). Broom: Convert Statistical Objects into Tidy Tibbles.
39. Muthén, L.K., and Muthén, B.O. (2017). Mplus: Statistical Analysis with Latent Variables: User's Guide (Version 8).
40. Tavakol, M., and Wetzel, A. (2020). Factor Analysis: a means for theory and instrument development in support of construct validity. Int. J. Med. Educ. *11*, 245–247. https://doi.org/10.5116/ijme.5f96.0f4a.
41. Howard, M.C. (2016). A Review of Exploratory Factor Analysis Decisions and Overview of Current Practices: What We Are Doing and How Can We Improve? Int. J. Hum. Comput. Interact. *32*, 51–62. https://doi.org/10.1080/10447318.2015.1087664.
42. Li, C.H. (2016). Confirmatory factor analysis with ordinal data: Comparing robust maximum likelihood and diagonally weighted least squares. Behav. Res. Methods *48*, 936–949. https://doi.org/10.3758/s13428-015-0619-7.
43. Hu, L.t., and Bentler, P.M. (1999). Cutoff criteria for fit indexes in covariance structure analysis: Conventional criteria versus new alternatives. Struct. Equ. Model.: A Multidiscip. J. *6*, 1–55. https://doi.org/10.1080/10705519909540118.
44. Hair, J.F., Black, W.C., Babin, B.J., and Anderson, R.E. (2013). Multivariate Data Analysis (Pearson Education Limited).
45. McCrae, R.R., Kurtz, J.E., Yamagata, S., and Terracciano, A. (2011). Internal consistency, retest reliability, and their implications for personality scale validity. Pers. Soc. Psychol. Rev. *15*, 28–50. https://doi.org/10.1177/1088868310366253.
46. Fonseca, C., Pettitt, J., Woollard, A., Rutherford, A., Bickmore, W., Ferguson-Smith, A., and Hurst, L.D. (2023). People with more extreme attitudes towards science have self-confidence in their understanding of science, even if this is not justified. PLoS Biol. *21*, e3001915. https://doi.org/10.1371/journal.pbio.3001915.
47. Lackner, S., Francisco, F., Mendonça, C., Mata, A., and Gonçalves-Sá, J. (2023). Intermediate levels of scientific

knowledge are associated with overconfidence and negative attitudes towards science. Nat. Hum. Behav. *7*, 1490–1501. https://doi.org/10.1038/s41562-023-01677-8.

48. Eysenbach, G., and Wyatt, J. (2002). Using the Internet for surveys and health research. J. Med. Internet Res. *4*, e13. https://doi.org/10.2196/jmir.4.2.e13.
49. Toscos, T., Drouin, M., Pater, J., Flanagan, M., Pfafman, R., and Mirro, M.J. (2019). Selection biases in technology-based intervention research: patients' technology use relates to both demographic and health-related inequities. J. Am. Med. Inform. Assoc. *26*, 835–839. https://doi.org/10.1093/jamia/ocz058.
50. Kruger, J., and Dunning, D. (1999). Unskilled and unaware of it: how difficulties in recognizing one's own incompetence lead to inflated self-assessments. J. Pers. Soc. Psychol. *77*, 1121–1134. https://doi.org/10.1037//0022-3514.77.6.1121.

HGGA, Volume 7

Supplemental information

Psychometric validation of the education and assessment of genetic literacy or the EAGL measure

Lily S. Barna, Yi Liao, Michael R. Wierzbicki, Gabriela M. Ramírez-Renta, Kimberly A. Kaphingst, and Chris Gunter

Supplementary Information Guide
Barna LS et al, “Psychometric Validation of the Education and Assessment of Genetic Literacy (EAGL) Measure”

Figure S1: Exploratory Factor Analysis (EFA) results for the EAGL-long, 3, 4, 5, and 9-factor solutions

Table S1. Participant Characteristics for Combined Sample (N = 2708), Split by Sample

Table S2: Comparison of Mean EAGL-Short Scores Across Three Survey Runs

Table S3: Complete Evaluation and Assessment of Genetic Literacy (EAGL-long) Instrument

Table S4: Factor Loadings and Communalities (h^2) for EAGL-long (Exploratory Factor Analysis, EFA)

Table S5: Five Factor EFA Table

Table S6. Four Factor EFA Table

Table S7: Eigenvalues and Variance Explained for Exploratory Factor Analysis of the EAGL-long

Table S8: Descriptive Statistics for Subjective Knowledge Items

Table S9: Frequency distribution for Knowledge Comprehension and Conceptual Knowledge items in CFA Sample (EAGL3, n = 1001)

Table S10: Average Scores Per Subscale for EAGL-long and EAGL-short

Table S11. Adjusted Mean Estimates for Each Subscale in EAGL-short

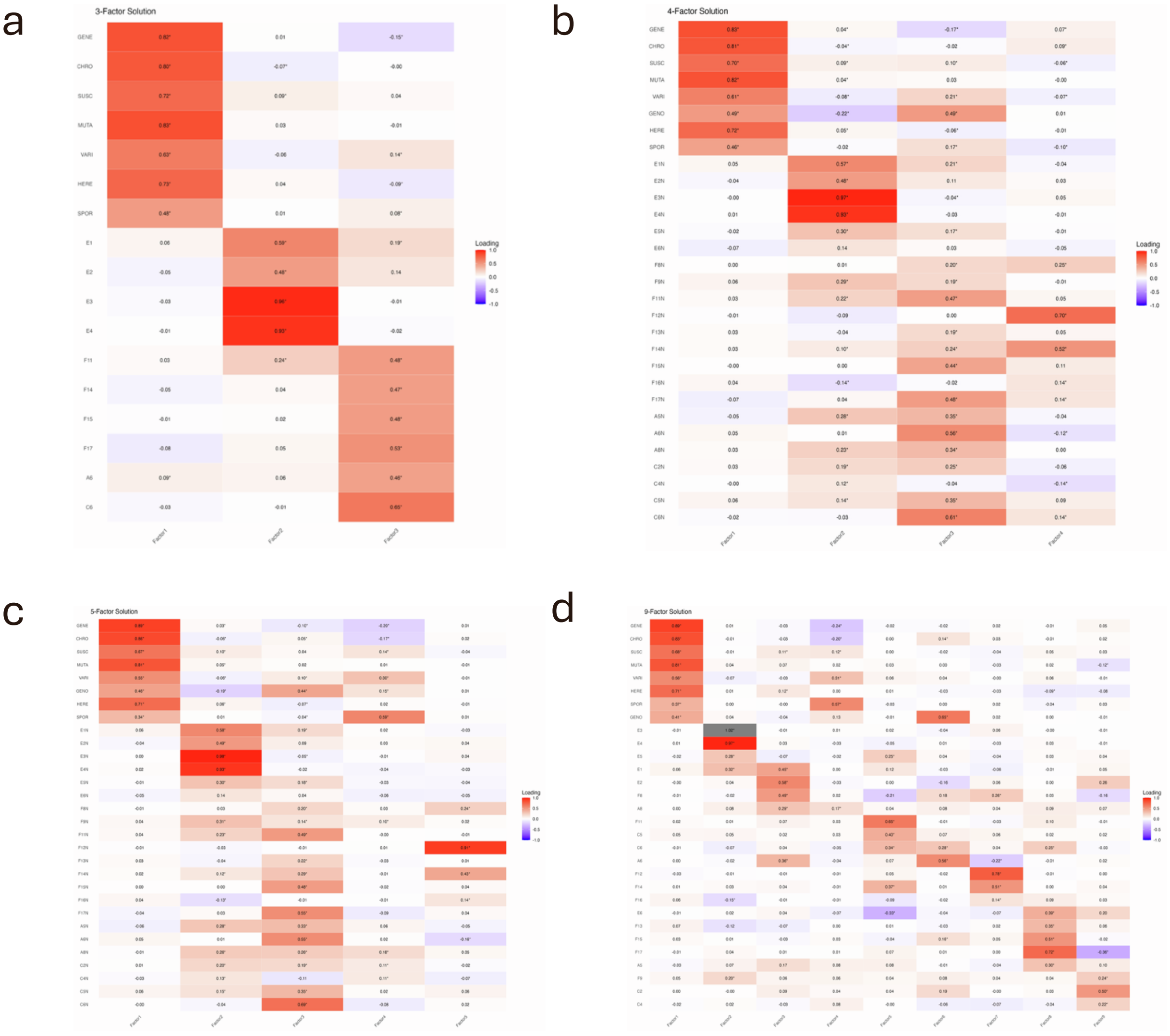

Figure S1. Exploratory Factor Analysis (EFA) results for the EAGL-long, 3, 4, 5, and 9-factor solutions. The heatmap displays factor loadings, with color intensity representing the magnitude of loadings (red for positive, blue for negative), and asterisks (*) indicating statistically significant loadings. Questions are grouped to highlight primary factor associations, with some items loading on multiple factors significantly, or loading on none. **(a)** 3-factor solution. **(b)** 4-factor solution. **(c)** 5-factor solution. **(d)** 9-factor solution contains nuanced splicing of clusters which illustrate multiple subject domains being tested. Loading values represent the strength of association between each item and the factor.

Table S1. Participant Characteristics for Combined Sample (N = 2708), Split by Sample				
Sample	Variable	Category	Frequency	% Total
Sample 1 (N = 1005)	Age	26-39	249	24.8%
		50-59	217	21.6%
		40-49	179	17.8%
		60-69	175	17.4%
		18-25	136	13.5%
		70+	49	4.9%
	Education	Bachelor's degree	398	39.6%
		High school diploma or equivalent	275	27.4%
		Professional degree	159	15.8%
		Associate degree	138	13.7%
		Doctorate degree	24	2.4%
		Some high school, no diploma	9	0.9%
		Kindergarten through 8th grade	2	0.2%
	Connection to Autism	No	822	81.8%
		Yes	183	18.2%
	Metro Status	Metro	896	89.15%
		Nonmetro	108	10.75
		Invalid Responses	1	0.10%
Sample 2 (n = 702)	Variable	Category	Frequency	% Total
	Age	18-25	134	19.1
		26-39	282	40.2
		40-49	142	20.2
		50-59	91	13.0
		60-69	43	6.1
		70+	10	1.4
	Education	High school diploma or equivalent	379	54.0%
		Associate degree	145	20.7%
		Bachelor's degree	95	13.5%
		Professional degree	41	5.8%
		Doctorate degree	22	3.1%
		Some high school, no diploma	18	2.6%
		Kindergarten through 8th grade	2	0.3%
	Connection to Autism	No	523	74.5%
		Yes	179	25.5%
	Metro Status	Metro	598	85.19%
		Nonmetro	102	14.53%
		Invalid Responses	2	0.28%

Sample 3 (n = 1001)	Variable	Category	Frequency	% Total
	Age	26-39	431	43.0%
		40-49	217	21.7%
		18-25	152	15.2%
		50-59	128	12.8%
		60-69	54	5.4%
		70+	19	1.9%
	Education	High school diploma or equivalent	441	44.0%
		Bachelor's degree	292	29.2%
		Associate degree	124	12.4%
		Professional degree	111	11.1%
		Doctorate degree	18	1.8%
		Some high school, no diploma	14	1.4%
		Kindergarten through 8th grade	1	0.1%
	Connection to Autism	No	791	79.0%
		Yes	210	21.0%
	Metro Status	Metro	880	87.91%
		Nonmetro	120	11.99%
		Invalid Responses	1	0.10%

Table S1. The table presents demographic characteristics across three EAGL samples comprising a total of 2,708 participants (Sample 1: N = 1,005; Sample 2: N = 702; Sample 3: N = 1,001). Age categories are presented in years and grouped into six ranges from 18-25 to 70+. Education levels are categorized from elementary education through doctoral degrees. "Connection to Autism" indicates whether participants reported having a personal connection to autism, either through themselves or through family members. Metro/non-metro distinctions were made via Federal Information Processing System (FIPS) Codes for States and Counties and Rural-Urban Continuum Codes (RUCC). A RUCC of 1-3 delineates a metropolitan area, with 4-9 delineating a nonmetropolitan county. The specific numbers indicate the level of adjacency to a metropolitan area, considering population of county and proximity to metro counties. Four participants had to be removed from analysis as they provided invalid 5-digit zip codes. Frequencies represent the number of participants in each category, and percentages are calculated based on the total sample size for each respective sample.

Table S2: Comparison of Mean EAGL-Short Scores Across Three Survey Runs					
Knowledge Domain	EAGL1 Mean (SD)	EAGL2 Mean (SD)	EAGL3 Mean (SD)	Overall Mean (SD)	Coefficient of Variance (CV) (%)
Subjective Knowledge	5.57 (0.98)	5.59 (0.97)	5.56 (0.98)	5.57 (0.97)	0.29
Applied Knowledge	7.05 (1.08)	6.89 (1.13)	6.93 (1.16)	6.96 (1.12)	1.19
Situational Knowledge	5.56 (1.12)	5.53 (1.20)	5.60 (1.19)	5.56 (1.15)	0.57
Knowledge Comprehension	5.47 (0.79)	5.44 (0.82)	5.33 (1.08)	5.41 (0.92)	1.35
Objective Knowledge	14.57 (1.81)	14.30 (1.91)	14.55 (1.88)	14.50 (1.86)	1.04

Table S2. The table presents the comparison of mean EAGL-short scores across the three survey runs. The mean score and standard deviation for each subscale of the EAGL-long are presented, along with the coefficient of variance.

Table S3: Complete Evaluation and Assessment of Genetic Literacy (EAGL-long) Instrument

First, we would like to see how familiar you are with words related to genetics.
For each word below, please rate how familiar you are with the word. For example, marking "Completely familiar" on the scale reflects that you are entirely familiar with the word, while marking "Not at all familiar" on the scale means that you are not familiar with the word in any capacity. Please select the answer that best reflects your view. Following that rating, you will then be presented with a fill-in-the-blank style question that utilizes a word related to genetics. Please do your best to select the appropriate response for the question, and please do not use the Internet or seek outside help in answering the questions.

Not at all familiar			Neither familiar nor unfamiliar			Completely familiar
1	2	3	4	5	6	7

A1_1. Genetic
Genetics is the study of how living things receive common traits from previous ____________.

a. generations **(C)**
b. experiences
c. examinations
d. d. achievements

A1_2. Chromosome
A chromosome contains ______________ material.

a. genetic **(C)**
b. digestive
c. cellular
d. d. brain

A1_3. Susceptibility

Susceptibility to a disease means you _________________ get the disease.

a. eventually will
b. definitely will
c. possibly will (C)
d. d. never will

A1_4 Mutation

A DNA mutation is ______________.

a. a type of cell
b. a type of virus
c. a change in your DNA sequence (C)
d. a measurement of DNA sequence

A1_5. Variation

Having a variation in the genetic code is __________________.

a. always harmful
b. can be harmful or benign (C)
c. always benign
d. extremely rare

A1_6. Genome

Your genome is _______________________.

a. where you make new DNA
b. all of the genes within one chromosome
c. your entire and complete set of DNA (C)
d. where cellular respiration occurs

A1_7. Heredity

Heredity is the transfer of characteristics from ____________________________.

a. the environment to the person
b. the sick to the healthy
c. the biological parent to their child (C)
d. the teacher to their student

A1_8. Sporadic
If someone is diagnosed with breast cancer without _____________ it is considered sporadic.

a. symptoms
b. a tumor
c. those around them knowing
d. a genetically increased likelihood (C)

A2_1. Please select the number 2 (two) from the list of numbers below:

a. 1
b. 4
c. 6
d. 2 (C)

B1_1. Imagine that we rolled a fair, six-sided die 1,000 times. Out of 1,000 rolls, how many times do you think the die would come up even (2, 4, or 6)?

a. 500 **(C)**
b. 250
c. 750

d. 800

B1_2. In the BIG BUCKS LOTTERY, the chances of winning a $10.00 prize is 1%. What is your best guess about how many people would win a $10.00 prize if 1,000 people each buy a single ticket to BIG BUCKS?

a. 100 people out of 1000
b. 50 people out of 1000
c. 10 people out of 1000 **(C)**
d. 1 person out of 1000

B1_3. Which of the following numbers represents the biggest risk of getting a disease?

a. 1 in 100
b. 1 in 10 **(C)**
c. 1 in 1000

C1_1. You are with your biological aunt, cousin, and sibling. You are asked to line them up by the amount of DNA, or genetic material, you share. You order them by most amount of DNA shared to least. That order is:

a. Aunt, cousin, sibling
b. Sibling, aunt, cousin **(C)**
c. Sibling, cousin, aunt
d. Cousin, sibling, aunt

C1_2. If both of my biological parents have brown eyes, what eye color could I have?

a. Only brown eyes
b. Brown or blue eyes
c. Brown, blue, or green eyes **(C)**
d. Only green eyes

C1_3. Certain genetic variants in the *BRCA* genes increase someone's risk for developing breast cancer. What is the most common purpose for obtaining *BRCA* genetic testing?

a. To determine if someone already has developed breast cancer
b. To determine if someone will never develop breast cancer
c. To determine if someone will one day develop breast cancer due to their environment
d. To determine if someone is genetically predisposed to developing breast cancer **(C)**

C1_4. If you have a genetic disease that was cured in your own red blood cells using gene therapy (replacing defective or missing genes with functional ones) as an adult, can you still pass on the disease to your biological children?

a. Yes, they could still get the disease **(C)**
b. No, they could not get the disease

C1_5. Cloning results in two organisms that are

a. genetically similar
b. genetically identical **(C)**
c. look alike but have different DNA
d. adults

C1_6. The term DNA stands for

a. Dynamic nitrogenous assembly
b. Double-nucleotide association
c. Deoxyribonucleic acid **(C)**
d. Downward nebula artifact

C1_7. When scientists say that a trait is "sex-linked," they mean that the genes related to the trait are

a. On the X or Y chromosome **(C)**
b. On all of the chromosomes
c. Only present in men
d. Only present in women

C2_1. Please select the third option from the list:

a. a
b. b
c. c **(C)**
d. d

D1_1. As far as you know, do you or does anyone in your immediate family have a genetic condition?

Yes …..1
No …...2

D1_2. As far as you know, have you or an immediate family member (e.g. parent, sibling, child) received any form of genetic testing?

Yes …..1
No …...2

[INFOGRAPHIC ABOUT GENETIC AND ENVIRONMENTAL CONTRIBUTORS TO AUTISM DIAGNOSIS HERE]

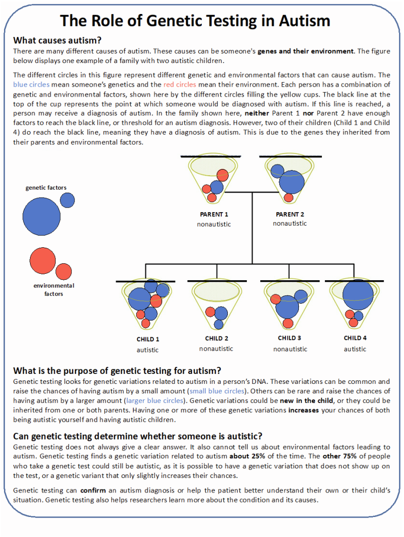

The Role of Genetic Testing in Autism

What causes autism?

There are many different causes of autism. These causes can be someone's **genes and their environment**. The figure below displays one example of a family with two autistic children.

The different circles in this figure represent different genetic and environmental factors that can cause autism. The blue circles mean someone's genetics and the red circles mean their environment. Each person has a combination of genetic and environmental factors, shown here by the different circles filling the yellow cups. The black line at the top of the cup represents the point at which someone would be diagnosed with autism. If this line is reached, a person may receive a diagnosis of autism. In the family shown here, **neither** Parent 1 **nor** Parent 2 have enough factors to reach the black line, or threshold for an autism diagnosis. However, two of their children (Child 1 and Child 4) do reach the black line, meaning they have a diagnosis of autism. This is due to the genes they inherited from their parents and environmental factors.

What is the purpose of genetic testing for autism?

Genetic testing looks for genetic variations related to autism in a person's DNA. These variations can be common and raise the chances of having autism by a small amount (small blue circles). Others can be rare and raise the chances of having autism by a larger amount (larger blue circles). Genetic variations could be **new in the child**, or they could be inherited from one or both parents. Having one or more of these genetic variations **increases** your chances of both being autistic yourself and having autistic children.

Can genetic testing determine whether someone is autistic?

Genetic testing does not always give a clear answer. It also cannot tell us about environmental factors leading to autism. Genetic testing finds a genetic variation related to autism **about 25%** of the time. The **other 75%** of people who take a genetic test could still be autistic, as it is possible to have a genetic variation that does not show up on the test, or a genetic variant that only slightly increases their chances.

Genetic testing can **confirm** an autism diagnosis or help the patient better understand their own or their child's situation. Genetic testing also helps researchers learn more about the condition and its causes.

[CHECKBOXES]
E1. What is the purpose of genetic testing for autism?

Genetic testing always provides a clear answer to whether or not someone is autistic ……..1
Genetic testing can tell you both the genetic and environmental causes of autism …..2
Genetic testing analyzes someone's DNA to find genetic variations related to autism …...3 **(C)**

E2. Please select the phrase that best completes the following statement: Genetic variations that could increase a person's chance of being autistic…

Can be present in some siblings and not others…..1 **(C)**
Are always the exact same between siblings…..2

[NUMERICAL TEXT BOX: RANGE 0-100]
E3. What percentage of individuals who have genetic testing for autism are found to have a variation related to autism?

Enter a number from 0–100: [ ]% **(25)**

[NUMERICAL TEXT BOX: RANGE 0-100]
E4. What percentage of individuals who have genetic testing for autism will receive results with no genetic variations related to autism?

Enter a number from 0–100: [ ]% **(75)**

E5. If neither of the biological parents are autistic, it is impossible for their child to be autistic.

True...1 False..2 **(C)**

E6. It is possible for a child to have a genetic variation that raises their chances of being autistic which neither of their biological parents have.

True...1 **(C)**
False..2

E7. Reaching the threshold for an autism diagnosis is due to a variable amount of environmental and genetic factors that vary per individual.

True...1 **(C)**
False..2

[TRUE/FALSE CHOICES]

**F1_1.** One can see a gene with the naked eye.	F
**F1_2.** Healthy parents can have a child with a hereditary condition.	T
**F1_3.** The onset of certain diseases is due to genes, environment, and lifestyle.	T
**F1_4.** A gene is a disease.	F
**F1_5.** The carrier of a gene linked to a disease may not have the disease themselves.	T
**F1_6.** All serious diseases are hereditary.	F
**F1_7.** Genes control hereditary characteristics.	T
**F1_8.** Genes are inside cells.	T
**F1_9.** The child of a carrier for a genetic condition is always also a carrier for the same condition.	F
**F1_10.** A gene is a piece of DNA.	T
**F1_11.** A gene is a cell.	F
**F1_12.** A gene is a part of a chromosome.	T

**F1_13.** Different genes are expressed in different body parts.	T
**F1_14.** Genes are bigger than chromosomes.	F
**F1_15.** The genome can be changed by human intervention.	T
**F1_16.** It is currently estimated that a person has about 20,000 genes.	T
**F1_17.** Environmental factors, such as UV radiation, can change our DNA sequence.	T

G1_1. What is the most important idea or fact about genetics that you feel you need to know? **[TEXTBOX RESPONSE]**

K1. How old are you?

< 18 1
18 - 25 2
26 - 39 3
40 - 49....................4
50 - 59....................5
60 - 69 6
70 +....................7

K2. What is the highest level of education you have completed?

No schooling completed…....................1
Kindergarten through 8th grade....................2
Some high school, no diploma 3
High school diploma or equivalent 4
Associate degree....................5
Bachelor's degree....................6
Professional degree....................7
Doctorate degree....................8

K3. Are you or is anyone in your immediate family autistic?

Yes 1
No 2

K4. What is the ZIP code of your primary residence? Enter a 5-digit ZIP Code. **[OPEN ENDED BOX]**

K5. Did you answer this survey honestly and to the best of your ability?

Yes 1
No 2

END

Sections:

A1-8 Likert-scale questions measure subjective knowledge
A1-8 multiple choice questions measure applied knowledge
B1-3 questions measure objective numeracy
C1-7 questions measure situational knowledge
E1-7 questions measure knowledge comprehension
F1-17 questions measure objective knowledge
G1 is an optional qualitative comment area
K1-K5 are demographics questions

Questions contained in the EAGL-short measure are shown in Figure 1 of the paper.

Table S4. Factor Loadings and Communalities (h^2) for EAGL-long (Exploratory Factor Analysis)										
Item	Factor1	Factor2	Factor3	Factor4	Factor5	Factor6	Factor7	Factor8	Factor9	h^2
GENE	0.886*	0.011	-0.03	-0.240*	-0.02	-0.022	0.017	-0.007	0.054	0.848
CHRO	0.833*	-0.014	-0.029	-0.204*	0	0.137*	0.034	-0.006	0.023	0.757
SUSC	0.681*	-0.01	0.110*	0.124*	0.002	-0.021	-0.039	0.054	0.029	0.497
MUTA	0.811*	0.037	0.065	0.017	0.028	0	-0.032	0.018	-0.123*	0.681
VARI	0.562*	-0.065	-0.034	0.309*	0.059	0.041	-0.004	0.055	-0.011	0.425
GENO	0.407*	0.035	-0.045	0.132	-0.009	0.648*	0.017	0.002	-0.009	0.607
HERE	0.709*	0.013	0.120*	0.001	0.011	-0.027	-0.026	-0.085	-0.08	0.532
SPOR	0.373*	0.002	-0.004	0.571*	-0.027	0	0.016	-0.042	0.027	0.469
E1	0.06	0.316*	0.448*	0	0.124	-0.027	-0.057	-0.013	0.049	0.326
E2	-0.002	0.038	0.582*	-0.031	0.002	-0.156	0.055	0	0.258	0.435
E3	-0.008	1.017*	-0.008	0.015	0.016	-0.038	0.063	-0.004	-0.01	1.040
E4	0.011	0.973*	0.025	-0.027	-0.049	0.015	-0.026	0.032	-0.005	0.953
E5	-0.024	0.283*	-0.069	-0.022	0.247*	0.039	-0.041	0.025	0.038	0.152
E6	-0.007	0.019	0.045	-0.065	-0.328	-0.039	-0.066	0.393*	0.2	0.315
F8	-0.005	-0.024	0.490*	0.024	-0.212	0.181	0.263*	0.026	-0.161	0.415
F9	0.046	0.195*	0.058	0.064	0.044	0.084	0.041	0.044	0.244*	0.120
F11	0.024	0.01	0.066	0.027	0.645*	-0.01	-0.031	0.096	-0.012	0.432
F12	-0.025	-0.014	-0.008	-0.012	0.052	-0.017	0.776*	-0.015	0.002	0.606
F13	0.066	-0.123	-0.072	0	0.008	-0.033	0.019	0.346*	0.058	0.149
F14	0.014	0.03	0.04	-0.013	0.368*	0.007	0.512*	0	0.037	0.402
F15	0.034	0.01	-0.028	0.027	-0.045	0.162*	0.053	0.512*	-0.024	0.297
F16	0.058	-0.150	-0.008	-0.011	-0.093	-0.022	0.143*	0.093	0.026	0.065
F17	-0.008	0.037	0.006	0.01	0.065	0.014	0.004	0.721*	-0.361*	0.656
A5	-0.03	0.066	0.174	0.08	0.079	-0.014	-0.045	0.300*	0.101	0.151
A6	0.001	-0.02	0.361*	-0.038	0.067	0.561*	-0.219	-0.011	0.023	0.500
A8	0.002	0.079	0.294*	0.171*	0.037	0.078	0.041	0.092	0.072	0.145
C2	0.002	-0.002	0.087	0.035	0.038	0.186	-0.002	0.026	0.503*	0.299
C4	-0.018	0.021	-0.029	0.078	-0.001	-0.064	-0.07	-0.042	0.224*	0.069
C5	0.046	0.05	0.022	0.028	0.399*	0.071	0.06	0.022	0.022	0.175
C6	-0.01	-0.067	0.041	-0.047	0.343*	0.281*	0.041	0.247*	-0.027	0.269

Table S4. This table presents the results of an exploratory factor analysis (EFA) for the EAGL-long instrument, displaying factor loadings and communalities across nine extracted factors (F1-F9). Each row represents a scale question. Factor loadings indicate the correlation between each item and the underlying factors, with values closer to ±1.0 representing stronger relationships. Asterisks (*) denote statistically significant factor loadings. The communality (h^2) represents the proportion of each item's variance explained by the factor solution, with values ranging from 0 to 1, where higher values indicate that more of the item's variance is accounted for by the extracted factors. Survey questions F1-F7 were excluded from this 9-factor solution due to excessively high accuracy rates, suggesting potential issues with model fit or interpretability.

Table S5. Five Factor EFA Table					
Variable	Factor1	Factor2	Factor3	Factor4	Factor5
GENE	0.891*	0.032*	-0.097*	-0.196*	0.007
CHRO	0.857*	-0.058*	0.052*	-0.173*	0.02
SUSC	0.666*	0.100*	0.038	0.142*	-0.035
MUTA	0.812*	0.049*	0.018	0.013	-0.01
VARI	0.546*	-0.059*	0.102*	0.305*	-0.006
GENO	0.455*	-0.194*	0.439*	0.149*	0.007
HERE	0.711*	0.056*	-0.069*	0.019	-0.012
SPOR	0.344*	0.011	-0.037*	0.586*	0.015
E1N	0.058	0.578*	0.187*	0.016	-0.028
E2N	-0.04	0.493*	0.088	0.028	0.044
E3N	0	0.976*	-0.054*	-0.012	0.045
E4N	0.019	0.925*	-0.024	-0.044	-0.028
E5N	-0.011	0.299*	0.181*	-0.032	-0.038
E6N	-0.048	0.138	0.041	-0.058	-0.053
F8N	-0.005	0.032	0.205*	0.025	0.236*
F9N	0.043	0.306*	0.141*	0.098*	0.021
F11N	0.038	0.226*	0.492*	-0.002	-0.009
F12N	-0.012	-0.032	-0.014	0.011	0.906*
F13N	0.033	-0.043	0.216*	-0.026	0.011
F14N	0.021	0.125*	0.290*	-0.011	0.431*
F15N	0.004	0.002	0.479*	-0.018	0.036
F16N	0.045	-0.134*	-0.01	-0.008	0.136*
F17N	-0.045	0.033	0.549*	-0.086	0.035
A5N	-0.063	0.284*	0.331*	0.058	-0.051
A6N	0.054	0.006	0.551*	0.017	-0.157*
A8N	-0.013	0.263*	0.257*	0.181*	0.049
C2N	0.013	0.205*	0.192*	0.108*	-0.022
C4N	-0.026	0.133*	-0.108	0.112*	-0.067
C5N	0.058	0.150*	0.348*	0.021	0.062
C6N	-0.002	-0.039	0.688*	-0.08	0.02

Table S5. Exploratory factor analysis showing factor loadings for EAGL-short scale items across five factors. Asterisks (*) indicate statistically significant loadings. Variables represent different dimensions of genetic knowledge and attitudes, with loadings ranging from -0.196 to 0.976.

Table S6. Four Factor EFA Table				
Variable	Factor1	Factor2	Factor3	Factor4
GENE	0.833*	0.044*	-0.166*	0.073*
CHRO	0.806*	-0.043*	-0.021	0.092*
SUSC	0.699*	0.086*	0.100*	-0.063*
MUTA	0.821*	0.041*	0.025	-0.004
VARI	0.609*	-0.080*	0.214*	-0.065*
GENO	0.488*	-0.216*	0.488*	0.012
HERE	0.722*	0.053*	-0.062*	-0.008
SPOR	0.456*	-0.02	0.167*	-0.096*
E1N	0.054	0.572*	0.213*	-0.039
E2N	-0.038	0.482*	0.114	0.03
E3N	-0.004	0.974*	-0.044*	0.049
E4N	0.007	0.930*	-0.027	-0.01
E5N	-0.022	0.303*	0.170*	-0.013
E6N	-0.066	0.144	0.029	-0.051
F8N	0.003	0.012	0.199*	0.248*
F9N	0.061	0.289*	0.194*	-0.013
F11N	0.034	0.224*	0.474*	0.054
F12N	-0.014	-0.086	0	0.700*
F13N	0.026	-0.039	0.190*	0.05
F14N	0.025	0.104*	0.239*	0.519*
F15N	-0.002	0.002	0.444*	0.106
F16N	0.045	-0.141*	-0.022	0.136*
F17N	-0.067	0.043	0.479*	0.135*
A5N	-0.054	0.280*	0.355*	-0.038
A6N	0.048	0.005	0.560*	-0.123*
A8N	0.025	0.235*	0.337*	0.003
C2N	0.032	0.188*	0.251*	-0.058
C4N	-0.004	0.122*	-0.036	-0.139*
C5N	0.059	0.140*	0.347*	0.09
C6N	-0.021	-0.026	0.607*	0.137*

Table S6. Exploratory factor analysis showing factor loadings for EAGL-short scale items across four factors. Asterisks (*) indicate statistically significant loadings. Variables represent different dimensions of genetic knowledge and attitudes, with loadings ranging from -0.166 to 0.974.

Table S7. Eigenvalues and Variance Explained for Exploratory Factor Analysis of the EAGL-long			
Factor	Eigenvalue	% of Variance	Cumulative %
Factor 1	3.744	12.48	12.50
Factor 2	2.268	7.56	20.0
Factor 3	1.096	3.65	23.70
Factor 4	0.615	2.05	25.70
Factor 5	1.099	3.66	29.4
Factor 6	0.984	3.28	32.7
Factor 7	1.044	3.48	36.2
Factor 8	1.257	4.19	40.4
Factor 9	0.680	2.27	42.60

Table S7. This table presents the eigenvalues and variance explained for each factor identified through Exploratory Factor Analysis of the EAGL-long measure. Each row represents a factor, ordered from highest to lowest eigenvalue. The first column presents the eigenvalues, or how much variance each factor explains. Those above the Kaiser criterion (eigenvalues > 1) are considered to be significantly meaningful. The second column presents this another way, through the percentage of total variance in the data that each factor accounts for individually. The third column, cumulative percentage, shows the total variance explained when you include each factor with the previous factors listed. All nine factors together explain 42.60% of the total variance in the data.

Table S8. Descriptive Statistics for Subjective Knowledge Items				
	Mean	SD	Skewness	Kurtosis
Genetic	6.09	0.99	-1.13	1.59
Chromosome	5.76	1.12	-0.86	0.99
Susceptibility	5.73	1.29	-1.25	1.87
Mutation	5.89	1.15	-1.24	2.07
Variation	5.38	1.46	-0.94	0.61
Heredity	6.1	1.04	-1.45	3.13
Sporadic	4.92	1.68	-0.66	-0.31
Overall	5.69	0.95	-0.69	0.48

Table S8. This table presents the descriptive statistics for the subjective knowledge items in the EAGL-short. Items were presented on a Likert scale, ranging from not at all familiar (0) to completely familiar (7). Mean scores, standard deviation, skewness, and kurtosis are presented.

Table S9. Frequency distribution for Knowledge Comprehension and Conceptual Knowledge items in CFA Sample (EAGL3, n = 1001)						
Factor	Item	1 (correct)	%	0 (incorrect)	%	n
Knowledge Comprehension	D1 (E1)	912	91.11	89	8.99	1001
	D2 (E2)	979	97.80	22	2.20	1001
	D3 (E3)	868	86.71	133	13.29	1001
	D4 (E4)	835	83.42	166	16.58	1001
Conceptual Knowledge	E1_1 (F11)	735	73.43	266	26.57	1001
	E1_2 (F14)	760	75.92	241	24.08	1001
	E1_3 (F15)	718	71.73	283	28.27	1001
	E1_4 (F17)	807	80.82	194	19.38	1001
	E2_1 (A6)	745	74.43	256	25.57	1001
	E2_2 (C6)	758	75.72	243	24.28	1001

Table S9. This table presents the frequency distributions for knowledge comprehensions and conceptual knowledge items within the sample used for CFA (EAGL3 sample, n = 1001). EAGL1 and EAGL2 were utilized for exploratory factor analysis (EFA) to identify the underlying factor structure, and EAGL3 for CFA to confirm the identified structure. This approach ensures a more rigorous validation of the factor model. Frequencies and percentages show the number and proportion of participants who answered each item correctly (1) or incorrectly (0). Items D1-D4 measure Knowledge Comprehension, while items E1_1-E2_2 measure Conceptual Knowledge as identified in exploratory factor analysis.

Table S10. Average Scores Per Subscale for EAGL-long and EAGL-short				
Instrument	Subscale	Mean	SD	Total Possible Score
EAGL-long	Subjective Knowledge	5.57	0.97	8
	Applied Knowledge	6.96	1.12	8
	Situational Knowledge	5.57	1.15	7
	Knowledge Comprehension	5.41	0.91	6
	Objective Knowledge	14.49	1.86	17
	Total Overall Score	38	3.86	46
EAGL-short	Subjective Knowledge	5.71	0.95	7
	Knowledge Comprehension	3.68	0.70	4
	Conceptual Knowledge	4.5	1.40	6
	Total Overall Score	13.89	2.01	17

Table S10. This table presents the average scores per subscale for both the EAGL-long and EAGL-short measures. The mean and standard deviation are presented for each subscale, along with for the overall score within each measure. The total possible scores are also presented.

Table S11. Adjusted Mean Estimates for Each Subscale in EAGL-short				
Subjective Knowledge	Variable	Value	Mean	Std. Err.
	Age	26-39	5.870501	0.048366
		40-49	5.956	0.055027
		50-59	5.886153	0.058928
		60-69	5.756377	0.069122
		70+	5.971	0.113743
		18-25	5.789449	0.06097
	Education	High school diploma or equivalent	5.725129	0.03996
		Associate degree	5.799588	0.054729
		Bachelor’s degree	5.836788	0.046494
		Professional degree	5.947521	0.062135
		Doctorate degree	5.953307	0.121663
		Less than High School	5.967149	0.143305
	Metro Status	Metro	5.821216	0.040745
		Nonmetro	5.921945	0.062807
	Connection to Autism	Yes	5.966647	0.056015
		No	5.776513	0.043638
Knowledge Comprehension	Age	26-39	3.643372	0.039485
		40-49	3.69233	0.043838
		50-59	3.732049	0.046419
		60-69	3.760242	0.053693
		70+	3.740388	0.085899
		18-25	3.65797	0.048235
	Education	High school diploma or equivalent	3.713923	0.031255
		Associate degree	3.713519	0.045621
		Bachelor’s degree	3.774669	0.039513
		Professional degree	3.552954	0.05662
		Doctorate degree	3.819741	0.117567
		Less than High School	3.651545	0.127593
	Metro Status	Metro	3.697003	0.034415
		Nonmetro	3.71178	0.049059
	Connection to Autism	Yes	3.720518	0.059353
		No	3.688265	0.034312
Conceptual Knowledge	Age	26-39	4.408883	0.087295
		40-49	4.322871	0.094327
		50-59	4.345177	0.100913
		60-69	4.303135	0.112935
		70+	4.442354	0.174413
		18-25	4.448759	0.102803
	Education	High school diploma or equivalent	4.432778	0.065618
		Associate degree	4.440231	0.104713
		Bachelor’s degree	4.656137	0.097307
		Professional degree	4.333828	0.156439
		Doctorate degree	4.630176	0.242011
		Less than High School	3.77803	0.333141
	Metro Status	Metro	4.501379	0.06269
		Nonmetro	4.255681	0.14664
	Connection to Autism	Yes	4.38015	0.095447
		No	4.37691	0.081367

Table S11. Supplementary table 11 presents the adjusted mean estimates for each demographic group across three subscales of the EAGL-short measure: subjective knowledge, knowledge

comprehension, and conceptual knowledge. Columns from left to right present the variables of interest, values of interests, adjusted means and standard error. Adjusted mean estimates control for the influence of other demographic variables on the variable of interest. Higher adjusted means indicate higher average scores within each subscale for the respective demographic group.
